# Public opinion on civil war in the USA as of mid-2024: findings from a nationally representative survey

**DOI:** 10.1186/s40621-025-00594-w

**Published:** 2025-07-03

**Authors:** Garen J. Wintemute, Yueju Li, Mona A. Wright, Andrew Crawford, Elizabeth A. Tomsich

**Affiliations:** 1 Department of Emergency Medicine, School of Medicine, UC Davis, 2315 Stockton Blvd, Sacramento, CA 95817 USA; 2 Violence Prevention Research Program, University of California Davis, Sacramento, CA USA; 3 California Firearm Violence Research Center, UC Davis, Sacramento, CA USA; 4 Department of Public Health Sciences, School of Medicine, UC Davis, Sacramento, CA USA

**Keywords:** Civil war, Political violence, Firearm violence, Violence and society, Authoritarianism, Racism, Domestic violent extremism, White supremacy, Christian nationalism, Militia movement, Boogaloo movement, Proud boys, Oath keepers, Three percenters, QAnon

## Abstract

**Background:**

In 2023, Wave 2 of an annual, nationally representative longitudinal survey found a concerning level of agreement that civil war was likely in the USA and, among those who agreed, widespread belief that civil war was needed. This study updates those findings to 2024 and explores respondents’ predicted involvement in such a conflict.

**Methods:**

Findings are from Wave 3, conducted May 23-June 14, 2024; participants were members of Ipsos KnowledgePanel. All respondents to prior waves who remained in KnowledgePanel were invited to participate; to facilitate comparison with 2023 findings, this analysis is restricted to Wave 3 respondents who had responded to both Waves 1 and 2. Outcomes are expressed as weighted proportions and adjusted prevalence differences.

**Results:**

The Wave 3 completion rate was 88.4% overall and 91.6% for respondents to Waves 1 and 2; there were 8185 respondents in the analytic sample. After weighting, half the sample was female (50.6%, 95% CI 49.1%, 52.1%); the weighted mean (SD) age was 50.8 (16.4) years. Few respondents agreed strongly or very strongly that “in the next few years, there will be civil war in the United States” (6.5%, 95% CI 5.7%, 7.3%) or that “the United States needs a civil war to set things right” (3.6%, 95% CI 3.0%, 4.2%). These prevalences were higher among subsets of respondents previously associated with increased support for and willingness to commit political violence. Of the small minority (3.7%, 95% CI 3.1%, 4.3%) who thought it very or extremely likely that they would be combatants, 44.5% (95% CI 36.5%, 52.6%) reported that they would convert to not likely if this were urged by family members; 23–31% were open to persuasion by friends, respected religious leaders, elected officials, and the media.

**Conclusions:**

In mid-2024, the expectation that civil war was likely and the belief that it was needed were uncommon and were unchanged from 2023. Those expecting to participate as combatants reported openness to change in response to input from many sources. These findings can help guide prevention efforts.

**Supplementary Information:**

The online version contains supplementary material available at 10.1186/s40621-025-00594-w.

## Background

Concerns about political violence in the United States (USA) extend to the possibility of widespread civil conflict [1–6]. In mid-2022, Wave 1 of our nationally representative longitudinal survey on political violence in the USA found that 13.7% of adults strongly or very strongly agreed with the statement that “in the next few years, there will be civil war in the United States” [7]. That prevalence fell to 5.7% with Wave 2 in 2023, but among those who agreed in Wave 2 that civil war was coming, 38.4% also strongly or very strongly agreed that “the United States needs a civil war to set things right” [8].

In 2023, both the expectation of and perceived need for civil war were higher among subgroups of respondents who were more likely than others to view political violence as justified, more willing to engage in political violence, and more likely to anticipate use of firearms for that purpose [9]. These groups included MAGA Republicans [10]; extreme conservatives [11]; persons who endorsed racist beliefs, the need for violence to effect social change, or specified extremist organizations and social movements [12]; and firearm owners who owned assault-type rifles, purchased firearms in 2020 or later, or carried loaded firearms in public all or nearly all the time [13]. 

Given the obvious need to monitor trends in these findings, and in advance of 2024’s nationwide elections, we repeated 2023’s items on civil war in 2024’s Wave 3 of the survey. Our prior expectation was that, because political polarization is increasing [2, 14, 15], expectation of and perceived need for civil war would be more prevalent in 2024 than in 2023. We added items regarding respondents’ support for right- and left-wing insurgency; their likely actions should large-scale conflict occur, including participating as a combatant and killing other combatants or non-combatants; and their openness to change on these matters. We report findings for the population as a whole and for the subsets mentioned previously.

## Methods

Methods for Wave 3 of this longitudinal survey closely followed those for Waves 1 and 2 [7–9]. Wave 3 was designed by the authors and administered online in English and Spanish from May 23 to June 14, 2024, by the survey research firm Ipsos [16]. The study was reviewed by the University of California Davis Institutional Review Board (protocol 187125: exempt from full review, category 2, survey research). The IRB waived a requirement for written or verbal consent. Before participants accessed the questionnaire, they were provided informed consent language that concluded, “[by] continuing, you are agreeing to participate in this study.” The study is reported following American Association for Public Opinion Research guidelines [17]. 

### Participants

Participants for Wave 1 were drawn from Ipsos KnowledgePanel, an online research panel that has been widely used in population-based research on violence and firearm ownership [18–23]. To establish a nationally representative panel, KnowledgePanel members are recruited on an ongoing basis through address-based probability sampling using data from the US Postal Service’s Delivery Sequence File [24, 25]. Recruitment into KnowledgePanel involves repeated contact attempts, if necessary, by mail and telephone. Recruited adults in households without internet access are provided a web-enabled device and free internet service, and a modest, primarily points-based incentive program seeks to encourage participation and promote participants’ retention in KnowledgePanel over time [24, 25]. 

A probability-proportional-to-size procedure was used to select a study-specific sample for Wave 1. All panel members who were aged 18 years and older were eligible for selection. Invitations were sent by e-mail; automatic reminders were delivered to non-respondents by e-mail and telephone beginning 3 days later [24, 25]. 

The Wave 1 survey was conducted May 13 to June 2, 2022. It included a main sample, which had a completion rate of 53% and provided the study population for our initial report [7], and oversamples of firearm owners, transgender people, combat veterans, and California residents that were recruited to ensure adequate statistical power for planned analyses. Compared with main sample nonrespondents, main sample respondents were older and more frequently white, non-Hispanic; were more often married; had higher education and income; and were less likely to be working [7]. 

Including the main sample and oversamples, Wave 1 comprised 12,947 respondents (see Supplement, Additional File 1: Figure S1, Table S1). Of those respondents, 11,140 (86.0%) remained active members of KnowledgePanel on Wave 2’s launch date in 2023 and were invited to participate in Wave 2. (The remaining 1807 Wave 1 respondents had left the cohort through normal attrition).

Wave 2 had 9385 respondents (completion rate of 84%), of whom 8932 (95.2%) remained active members of KnowledgePanel on Wave 3’s launch date in 2024 and were invited to participate in Wave 3 (see Supplement, Additional File 1: Figure S1, Table S1). Another 453 Wave 2 respondents had left the cohort through normal attrition.

Invitations to participate in Wave 3 were also sent to 1132 Wave 1 respondents who had not responded to Wave 2 but remained active members of KnowledgePanel on Wave 3’s launch date (see Supplement, Additional File 1: Figure S1, Table S1). To facilitate comparison with the Wave 2 findings on civil war, eligibility for this analysis was restricted to Wave 3 respondents who had responded to both Wave 1 and Wave 2.

A final Wave 3 survey weight variable provided by Ipsos adjusted for the initial probability of selection into KnowledgePanel and for survey-specific nonresponse and over- or under-coverage using design weights with post-stratification raking ratio adjustments. As with prior samples, the weighted 2024 sample is designed to be statistically representative of the noninstitutionalized adult population of the US as reflected in the 2021 March supplement of the Current Population Survey [24, 25]. 

### Measures

Sociodemographic data were collected by Ipsos from profiles created and maintained by KnowledgePanel members. Our primary measures of interest concerned respondents’ views on a potential civil war in the US. As in Wave 2, participants were asked about their agreement with the following statements: “In the next few years, there will be civil war in the United States,” and “The United States needs a civil war to set things right.”

New items in the Wave 3 survey asked participants whether, in the event of a right-wing (or, separately, a left-wing) anti-government insurgency, they would most likely support the insurgency, the government, or neither side. They were asked how likely they would be to act in specified ways if a civil war did occur; these actions ranged from leaving the USA to killing others as a combatant. Finally, participants who thought it either not likely or very or extremely likely that they would participate as a combatant were asked whether they would change their position if urged to do so by family members, friends, religious leaders, and others.

Our analysis included survey items (from Wave 3 unless specified otherwise here) that addressed respondent social characteristics in 5 broad domains: political party affiliation and political ideology, support for authoritarianism, beliefs about race and ethnicity and American society (Wave 2), beliefs about the potential need for violence to effect social change in the USA, firearm ownership and use (Wave 1), and approval of specified extreme right-wing political organizations and social movements (Wave 1; organizations were the Proud Boys, Oath Keepers, and Three Percenters; social movements were the QAnon, white supremacy, Christian nationalist, militia, and boogaloo movements). We included items from Wave 3 on respondents’ willingness to engage in political violence at varying levels of severity and their expectations of their use of firearms for political violence. Further information on the construction of these measures has been published previously [7–13] and is provided in the Supplement (see Additional File 1), as are the full text of all questions reported on here and sources for questions from surveys by other investigators.

### Implementation

Ipsos translated the questionnaire into Spanish, and interpreting services staff at UC Davis Medical Center reviewed the translation. Twenty-three KnowledgePanel members participated in a pretest of the English language version that was administered May 10–14, 2024.

Respondents were randomized 1:1 to receive response options in order from either negative to positive valence (example: from ‘do not agree’ to ‘strongly agree’) or the reverse throughout the questionnaire. Where a question presented multiple statements for respondents to consider, the order in which those statements were presented was randomized unless ordering was necessary. Logic-driving questions (those to which responses might invoke a skip pattern) included non-response prompts.

We employed unipolar response arrays without a neutral midpoint (e.g., do not agree, somewhat agree, strongly agree, very strongly agree). The literature is not in agreement on whether such midpoints should be included [26, 27]. We were persuaded by the studies reviewed by Chyung et al., [26] which suggest that such midpoints allow respondents to choose “a minimally acceptable response as soon as it is found, instead of putting effort to find an optimal response,” a behavior known as satisficing. According to those authors, satisficing is particularly common when respondents are uncomfortable with the topics of the survey or under social desirability pressures; both conditions apply here.

### Statistical analysis

Analyses were conducted using SAS version 9.4 (SAS Institute, Inc., Cary, NC). To generate prevalence estimates, we calculated weighted percentages and 95% confidence intervals (CI) using PROC SURVEYFREQ. A Spearman correlation coefficient was calculated using PROC CORR.

To compute adjusted prevalence differences and 95% CIs, we defined outcomes dichotomously and used PROC SURVEYREG, employing robust standard errors to correct for design effects and heteroskedasticity in binary outcomes. We employed the same model used in the Wave 2 analysis [9]. That model included age, race and ethnicity, gender, education, income, Census division, and rurality.

P-values were corrected for multiple comparisons by controlling the false discovery rate using the Benjamini-Hochberg method [28]. The resulting values are known as FDR-adjusted (or FDR-corrected) p-values or as q-values [29]; we employ the latter term here. Q-values represent the probability that the given difference would be a false discovery; they represent the expected proportion of “false positives” that would be seen among the collection of all differences whose q-values were at or below the given q-value.

The survey was in the field when Donald Trump was convicted on 34 felony charges in New York State Supreme Court at approximately 5 PM Eastern Daylight Time on May 30, 2024 [30]. We added a sensitivity analysis comparing responses on selected items submitted before and after the convictions were announced.

## Results

For the 2024 Wave 3 survey overall, 10,064 invitations were sent and 8896 individuals completed the survey; the overall completion rate was 88.4%. Of the 8932 Wave 2 respondents who were invited to participate, 8185 completed the survey, yielding a 91.6% completion rate for this analysis (see Supplement, Additional File 1, Figure S1). The overall median survey completion time was 22 min (interquartile range, 15.7 min). Item non-response in this analysis ranged from 0.05 to 3.5%, and 3 items had non-response percentages above 3.0% (see Supplement, Additional File 1).

After weighting, half of the respondents (50.6%, 95% CI 49.1%, 52.1%) were female; 63.3% (95% CI 61.8%, 64.9%) were white, non-Hispanic (see Supplement, Additional File 1, Table S2). The weighted mean (SD) respondent age was 50.8 (16.4) years.

Few respondents (6.5%, 95% CI 5.7%, 7.3%) agreed strongly or very strongly with the proposition that “in the next few years, there will be civil war in the United States” or that “the United States needs a civil war to set things right” (3.6%, 95% CI 3.0%, 4.2%) (Additional file 2, Table 1).

Responses to these 2 items were correlated (Spearman correlation coefficient = 0.46; *p* = 0.001); among respondents who strongly or very strongly agreed that civil war was coming, 36.0% (95% CI 29.7%, 42.3%) strongly or very strongly agreed that it was needed (Additional file 3, Table 2).

Insurgency received little support; 8.0% (95% CI 7.3%, 8.8%) of respondents would support a right-wing anti-government insurgency, and 6.7% (95% CI 5.9%, 7.5%) would support a left-wing insurgency (Additional file 2, Table 1). In either case, most respondents would support neither side, and about 1 in 3 would support the government.

If large-scale conflict did occur, 47.9% (95% CI 46.4%, 49.3%) of respondents would “sit it out” in the United States, without participating (Additional file 2, Table 1). About 1 in 8 (12.4%, 95% CI 11.3%, 13.4%) thought it very or extremely likely that they would leave the country.

Participants were asked directly about their participation as a combatant in a large-scale conflict. A large majority (84.2%, 95% CI 83.1%, 85.3%) considered this not likely (Additional file 2, Table 1). About 1 in 30 (3.7%, 95% CI 3.1%, 4.3%) thought this very or extremely likely, and a similar percentage (3.4%; 95% CI 2.9%, 4.0%) thought it very or extremely likely that they would kill a combatant from the opposing side.

Respondents who thought it unlikely that they would participate as combatants were, with few exceptions, not open to changing their position (Additional file 2, Table 1). But of respondents who considered participation as a combatant to be very or extremely likely, 44.5% (95% CI 36.5%, 52.6%) reported that their position would convert to “not likely” if this were urged by family members, 23.4% (95% CI 16.7%, 30.1%) if urged by friends, 30.5% (95% CI 23.0%, 38.0%) if by a respected religious leader, 26.3% (95% CI 19.4%, 33.3%) if by a respected elected official or other public figure, and 23.6% (95% CI 16.4%, 30.8%) if by a respected news or social media source. Findings were similar for respondents who additionally reported that it was very or extremely likely that they would “kill a combatant from the opposing side” (Additional file 2, Table 1).

### Variation among subgroups

This section and Table 3 (Additional file 4) summarize findings for subgroups on 4 key measures: the expectation that civil war was coming, the belief that it was needed, support for a right- or left-wing insurgency, and willingness to participate as a combatant; additional detail is presented in the Supplement (see Supplement, Additional File 1, Tables S3-S13).

Prevalences for all 4 measures (including right-wing but not left-wing insurgency) were higher among strong Republicans, MAGA Republicans, non-Republican members of the MAGA movement, and extreme conservatives, than among their respective comparison groups (Additional file 4, Table 3; see Supplement, Additional File 1, Tables S3-S5). Strong Democrats and extreme liberals had higher prevalences only of support for a left-wing insurgency (Additional file 4, Table 3; see Supplement, Additional File 1, Tables S3-S5).

Prevalences for all 4 measures (including right-wing but not left-wing insurgency) were also higher among respondents who strongly or very strongly agreed with statements expressing authoritarianism (Additional file 4, Table 3; see Supplement, Additional File 1, Table S6), and those in strong or moderate agreement with racist beliefs (Additional file 4, Table 3; see Supplement, Additional File 1, Table S7), than among their comparators. Prevalences were substantially higher among respondents who strongly agreed with statements of the potential need for violence to effect social change than among those who did not agree (Additional file 4, Table 3; see Supplement, Additional File 1, Table S8). The same was true for respondents who strongly approved of the specified extreme right-wing political organizations and social movements, among whom more than half expected civil war (55.7%, 95% CI 37.0%, 74.5%) and thought that it was needed (50.8%, 95% CI 31.9%, 69.7%), and 39% (95% CI 20.6%, 57.4%) thought it very or extremely likely that they would participate as combatants (Additional file 4, Table 3; see Supplement, Additional File 1, Table S9).

Differences on these measures between firearm owners and non-owners without firearms at home were modest, where they existed (Additional file 4, Table 3; see Supplement, Additional File 1, Table S10). Owners of assault-type rifles more often expected to serve as combatants than did those who owned only handguns (Additional file 4, Table 3; see Supplement, Additional File 1, Table S11), and prevalences for all 4 measures were higher among those who purchased firearms recently (Additional file 4, Table 3; see Supplement, Additional File 1, Table S12) and those who carried firearms in public all or nearly all the time (Additional file 4, Table 3; see Supplement, Additional File 1, Table S13).

Across all subgroups, respondents who thought it very or extremely likely that they would participate as combatants were much more open to change if urged to do so by family members and others than were respondents who thought combat unlikely; differences were comparable to those for the population as a whole. Counts of responses for likely combatants were small, however, and within-subgroup differences were generally not significant (data not shown).

### Variation with willingness to commit political violence and use firearms

Respondents who were very or completely willing to damage property, threaten a person, or kill a person to advance political objectives were substantially more likely than unwilling respondents to agree that civil war was coming and that it was needed and to predict that they would participate as a combatant (Additional file 5, Table 4). They were generally not more likely to support an insurgency.

Prevalences for all 4 measures were higher among respondents who thought it very or extremely likely that they would use firearms in a future situation where they considered political violence justified, as compared with respondents who thought it not likely, except that respondents who thought it likely that they would shoot someone did not have higher prevalences of support for an insurgency (Additional file 6, Table 5).

### Sensitivity analysis

There were no significant differences between responses submitted before or after the Trump convictions in prevalences of respondents strongly or very strongly agreeing that civil war was coming or that it was needed, and no differences in prevalences of respondents reporting that they would support a right- or left-wing insurgency (data not shown).

## Discusssion

In this nationally representative longitudinal survey, strong or very strong agreement with statements that civil war was coming and that it was needed was reported by only 6.5% and 3.6% of respondents, respectively. There was no change from 2023, when corresponding prevalences were 5.7% and 3.8%.^9^ These findings are contrary to our expectation that both would increase as political polarization increased during this election year [2, 14, 15, 31]. They are concordant with the fact that there was no increase in firearm purchasing in 2024 beyond long-term trends [32]. Such an increase did occur prior to and following political violence in 2021 [33], and others have found that 24% of firearm purchases in 2023 were “motivated by concerns of political violence.” [34] An increase might therefore have been expected if individuals were purchasing firearms in anticipation of participating in political violence in 2024. (The observed absence of an increase, which has extended into 2025, should not be taken to mean that such purchases are not occurring).

While experts consider formal civil war to be highly unlikely, predictions are less optimistic for sporadic outbreaks of political violence, targeted attacks intended to disrupt the electoral process, and insurgency [1–6, 15, 31, 35]. We found little support for an insurgency, and only 3.7% of respondents thought it very or extremely likely that they would participate in civil conflict as combatants. Pluralities or majorities of respondents reported that, in the event of insurgency or civil war, they would be neutral nonparticipants.

As in 2023 [9], there was important variation among respondents, with prevalences generally being higher among subsets that have also been found to be more likely than others to view political violence as justified and frequently more willing than others to engage in such violence themselves [10–13]. Prevalences were also higher among respondents who had previously indicated their willingness to participate in political violence and to use firearms in such violence.

One set of findings appears to provide specific guidance for intervention to prevent large-scale political violence. Respondents who considered themselves very or extremely likely to participate as combatants reported a high degree of openness to change; 44.5% believed they would become unlikely to participate if urged in that direction by family members, and smaller but still important proportions were open to persuasion by other sources, including respected public figures and news and social media sources. This finding substantiates our prior inference [9] that a climate of non-support for political violence may reduce the likelihood that it will occur. It is in accord with findings by others, particularly regarding the beneficial influence of statements by public officials condemning political violence [2, 15, 36]. 

Our findings suggest, however, that the most efficacious messengers at the individual level are the 84% of Americans who are not likely to participate as combatants, assuming they have family members or friends who believe otherwise. Complementary strategies that rely on trusted community leaders and the media can readily be developed and might be most efficacious at the population level, given the reach of those sources of information. It is not unreasonable to expect effects of such interventions to generalize beyond participation in large-scale conflict to participation in political violence of other types.

William Haddon, Jr., whose work laid the foundation for modern injury epidemiology, repeatedly emphasized that attempts to break a causal chain ending in an adverse event should focus on the link in that chain that is most easily broken [37, 38]. Political violence arises in large part from deeply-held attitudes and beliefs (such as racism, sexism, and xenophobia) that shape individual identity, and from affective political polarization [2, 14, 15]. But those attitudes and beliefs resist change, and interventions on polarization may not produce long-lasting changes in behavior [2, 14, 15]. The findings of this study suggest that for a substantial proportion of high-risk individuals the primary strategy, at least in the short run, should be to uncouple identity and polarization from their adverse consequence—participation in political violence.

That suggestion is supported by other basic tenets of the public health approach to prevention. Violence is a health problem, and participation in violence is therefore a health behavior. A variety of health behaviors are open to influence by family members [39], friends [40], coworkers [41], social media contacts [42], and well-known public figures [43]. Haddon would categorize an intervention relying on these sources of influence as making pre-event changes in the social environment [37, 38]. 

Such a strategy would not be new to political violence prevention. As 2 leading political violence researchers noted in a 2024 review, “interpersonal influence by trusted friends and family members is generally the strongest persuasive force in politics.” [15] But efforts at suasion would likely not affect the would-be combatants who, our data suggest, are not open to change regarding their expected participation in violence. An array of complementary approaches will be necessary for them [44–47]. Among these are traditional threat assessment and law enforcement investigative efforts, but here, too, there is a role for individual members of the public to play, by relaying threats to commit political violence to professionals who can intervene [48, 49]. 

### Limitations

Several limitations exist. The findings are for a single year. They are subject to sampling error and inattentive or strategic responses. Non-response bias is a concern; respondents and nonrespondents differed in age and gender, which are related to support for political violence. Arguably, nonresponse was most important in Wave 1; response rates for Waves 2 and 3 were high. Nonetheless, serial selection over 3 waves of the survey might lead to underestimation of true prevalences on some measures (such as expectation of and perceived need for civil war and predicted participation as a combatant) and overestimation of others (such as openness to change with regard to participation as a combatant). Our outcome measures include reported support for and willingness to engage in political violence, which are imperfect predictors of actual commission of political violence. Some outcomes are uncommon; this was particularly the case for openness to change among subsets of respondents who thought it likely that they would participate in combat. Questions on specific actions respondents might take during large-scale conflict did not specify the form of that conflict.

## Conclusion

Findings from this large, nationally representative longitudinal survey indicate that expectations of and support for civil war were uncommon in mid-2024 and little changed from 2023. Among respondents who thought it likely that they would participate as combatants, large percentages reported that they would abandon this position if urged to do so by family members, friends, respected leaders, and the media. These findings have direct implications for prevention efforts.

## Electronic supplementary material

Additional file 1: Supplement: Public Opinion on Civil War in the USA as of Mid-2024: Findings from a Nationally Representative Survey Description of data: Questions that supplied data for this analysis, supplemental methods text, 1 figure, and 13 tables

Additional file 2: Table 1. Beliefs and predictions regarding civil war in the USA Description of data: Data for manuscript table 1. 

Additional file 3: Table 2. Relationship between expectations of and perceived need for civil war in the USA Description of data: Data for manuscript table 2. 

Additional file 4: Table 3. Summary of beliefs and predictions regarding civil war in the USA for subgroups of respondents Description of data: Data for manuscript table 3. 

Additional file 5: Table 4. Engagement in political violence and beliefs and predictions regarding civil war in the USA Description of data: Data for manuscript table 4

Additional file 6: Table 5. Firearm possession and use and beliefs and predictions regarding civil war in the USA Description of data: Data for manuscript table 5

## Data Availability

The datasets generated and/or analyzed during the current study are not publicly available as analyses are continuing but will be made available to qualified researchers subject to the terms of a data use agreement.

## Abbreviations

APD: Adjusted prevalence difference
CI: Confidence interval
SD: Standard deviation

## References

[1] Walter BF. How civil wars start and how to stop them. New York: Crown; 2022.

[2] Kleinfeld R. Polarization, democracy, and political violence in the United States: what the research says. Carnegie Endowment for International Peace. 2023. https://carnegieendowment.org/2023/09/05/polarization-democracy-and-political-violence-in-united-states-what-research-says-pub-90457. Accessed 24 June 2025.

[3] Kleinfeld R. There won’t be a civil war… but the Far right is pushing politics toward a darker and more violent future. Persuasion. 2022. https://www.persuasion.community/p/there-wont-be-a-civil-war. Accessed 24 June 2025.

[4] Gale WG, West DM. Is the US headed for another civil war? Brookings institution. 2021. https://www.brookings.edu/articles/is-the-us-headed-for-another-civil-war/. Accessed 24 June 2025.

[5] Simon S, Stevenson J. The threat of civil breakdown is real. Politico. 2023. https://www.politico.com/news/magazine/2023/04/21/political-violence-2024-magazine-00093028. Accessed 24 June 2025.

[6] Walter BF, Marche S, Parker CS. ‘These are conditions ripe for political violence’: how close is the US to civil war? The Guardian. 2022. https://www.theguardian.com/us-news/2022/nov/06/how-close-is-the-us-to-civil-war-barbara-f-walter-stephen-march-christopher-parker. Accessed 24 June 2025.

[7] Wintemute GJ, Robinson SL, Crawford A, Tancredi D, Schleimer JP, Tomsich EA, Reeping PM, Shev AB, Pear VA. Views of democracy and society and support for political violence in the USA: findings from a nationally representative survey. Inj Epidemiol. 2023;10(1):45. 37770994 10.1186/s40621-023-00456-3 PMC10540371

[8] Wintemute GJ, Robinson SL, Crawford A, Tomsich EA, Reeping PM, Shev AB, Velasquez B, Tancredi D. Single-year change in views of democracy and society and support for political violence in the USA: findings from a 2023 nationally representative survey. Inj Epidemiol. 2024;11(1):20. 38773542 10.1186/s40621-024-00503-7 PMC11110245

[9] Wintemute GJ, Li Y, Velasquez B, Crawford A, Reeping PM, Tomsich EA. Expectations of and perceived need for civil war in the united states: findings from a 2023 nationally representative survey. Inj Epidemiol. 2024;11(1):40. 39210500 10.1186/s40621-024-00521-5 PMC11360528

[10] Wintemute GJ, Robinson R, Tomsich EA. MAGA republicans’ views of American democracy and society and support for political violence: findings from a nationwide population-representative survey. PLoS ONE. 2024;19(1):e0295747. 38170700 10.1371/journal.pone.0295747 PMC10763974

[11] Wintemute GJ, Crawford A, Robinson A, Schleimer JP, Tomsich EA, Pear VA. Party affiliation, political ideology, views of American democracy and society, and support for political violence: findings from a nationwide population-representative survey. SocArXiv. 2022. 10.31235/osf.io/n9b36. Accessed 24 June 2025.

[12] Wintemute GJ, Velasquez B, Li Y, Tomsich EA, Reeping PM, Robinson SL. Racist and pro-violence beliefs, approval of extreme right-wing political organizations and movements, and support for political violence in the united States. SocArXiv. 2023. 10.31235/osf.io/c9vtr 10.1186/s40621-025-00631-8 PMC12683887 41361497

[13] Wintemute GJ, Crawford A, Robinson S, Tomsich E, Reeping P, Schleimer J, Pear V. Firearm ownership and support for political violence in the united States. JAMA Netw Open. 2024;7(4):e243623. 38592725 10.1001/jamanetworkopen.2024.3623 PMC11004826

[14] Kalmoe NP, Mason L. Radical American partisanship. Chicago: University of Chicago Press; 2022.

[15] Mason L, Kalmoe N. How to prevent a spiral of political violence in America. Foreign Aff. 2024. https://www.foreignaffairs.com/united-states/how-prevent-spiral-political-violence-america. Accessed 24 June 2025.

[16] Ipsos. 2024. https://www.ipsos.com/en. Accessed 24 June 2025.

[17] American Association for Public Opinion Research. Code of professional ethics and practices. (April 2021 edition). https://aapor.org/standards-and-ethics/. Accessed 24 June 2025.

[18] Kravitz-Wirtz N, Aubel A, Schleimer J, Pallin R, Wintemute G. Public concern about violence, firearms, and the COVID-19 pandemic in California. JAMA Netw Open. 2021;4(1):e2033484. 33394004 10.1001/jamanetworkopen.2020.33484 PMC7783542

[19] Wintemute GJ, Aubel AJ, Pallin RS, Schleimer JP, Kravitz-Wirtz N. Experiences of violence in daily life among adults in california: a population-representative survey. InjEpidemiol. 2022;9:1. 10.1186/s40621-021-00367-1. 10.1186/s40621-021-00367-1 PMC8721630 34980276

[20] Schleimer J, Kravitz-Wirtz N, Pallin R, Charbonneau A, Buggs S, Wintemute G. Firearm ownership in california: a latent class analysis. Inj Prev. 2020;26(5):456–62. 31601624 10.1136/injuryprev-2019-043412

[21] Miller M, Zhang W, Azrael D. Firearm purchasing during the covid-19 pandemic: results from the 2021 National firearms survey. Ann Intern Med. 2022;175(2):219–25. 34928699 10.7326/M21-3423 PMC8697522

[22] Miller M, Azrael D. Firearm storage in US households with children: findings from the 2021 National firearm survey. JAMA Netw Open. 2022;5(2):e2148823. 35191973 10.1001/jamanetworkopen.2021.48823 PMC8864510

[23] Salhi C, Azrael D, Miller M. Patterns of gun owner beliefs about firearm risk in relation to firearm storage: a latent class analysis using the 2019 National firearms survey. Inj Prev. 2020:injuryprev-2019-043624. 10.1136/injuryprev-2019-043624 32665253

[24] Ipsos. KnowledgePanel^®^: a methodological overview. 2020. https://www.ipsos.com/sites/default/files/ipsosknowledgepanelmethodology.pdf. Accessed 24 June 2025.

[25] Ipsos. KnowledgePanel sampling and weighting methodology. 2015. https://www.ipsos.com/sites/default/files/kpsamplingandweighting.pdf. Accessed 24 June 2025.

[26] Chyung SY, Roberts K, Swanson I, Hankinson A. Evidence-based survey design: the use of a midpoint on the likert scale. Perform Improv. 2017;56(10):15–23.

[27] Westwood SJ, Grimmer J, Tyler M, Nall C. Current research overstates American support for political violence. PNAS. 2022;119(12):e2116870119. 35302889 10.1073/pnas.2116870119 PMC8944847

[28] Benjamini Y, Hochberg Y. Controlling the false discovery rate: a practical and powerful approach to multiple testing. J R Statist Soc B. 1995;57(1):289–300.

[29] Storey JD. The positive false discovery rate: a bayesian interpretation and the q-value. Ann Statist. 2003;31(6):2013–35.

[30] Gamio L, Yourish K, Haag M, Bromwich JE, Haberman M, Lai KKR. The Trump Manhattan criminal verdict, count by count. N Y Times. 2024 May 30. https://www.nytimes.com/interactive/2024/05/30/nyregion/trump-hush-money-verdict.html. Accessed 24 June 2025.

[31] Armed Conflict Location & Event Data Project (ACLED). Will Past US Election Turbulence Strike Again in 2024? 2024 Mar 14. https://acleddata.com/2024/03/14/will-past-us-election-turbulence-strike-again-in-2024/. Accessed 24 June 2025.

[32] Federal Bureau of Investigation. NICS firearm checks by month year state type—last 5 years. 2024. https://www.fbi.gov/file-repository/nics_firearms_checks_-_month_year_by_state_type-last-5-years.pdf/view. Accessed 24 June 2025.

[33] Wintemute GJ. Guns, violence, politics: the Gyre widens. Inj Epidemiol. 2021;8(1):64. 34727981 10.1186/s40621-021-00357-3 PMC8562372

[34] Valek R, Ward JA, Jones V, Crifasi CK. Political violence, Racial violence, and new gun ownership: results from the 2023 National survey of gun policy. Inj Epidemiol. 2024;11:48. 39243093 10.1186/s40621-024-00527-z PMC11378614

[35] Federal Bureau of Investigation and Department of Homeland Security. Strategic intelligence assessment and data on domestic terrorism. 2023. https://www.fbi.gov/file-repository/fbi-dhs-domestic-terrorism-strategic-report.pdf. Accessed 24 June 2025.

[36] Voelkel JG, Stagnaro MN, Chu J et al. Megastudy identifying effective treatments to strengthen americans’ Democratic attitudes. 2023. [Preprint.]. https://osf.io/preprints/osf/y79u5. Accessed 24 June 2025.

[37] Haddon W Jr. On the escape of tigers: an Ecologic note. Am J Public Health. 1970;60(12):2229–34. 10.2105/ajph.60.12.2229-b PMC1349282 5530409

[38] Haddon W Jr. Advances in the epidemiology of injuries as a basis for public policy. Public Health Rep. 1980;95(5):411–21. 7422807 PMC1422748

[39] Michaelson V, Pilato KA, Davison CM. Family as a health promotion setting: A scoping review of conceptual models of the health-promoting family. PLoS ONE. 2021;16(4):e0249707. 33844692 10.1371/journal.pone.0249707 PMC8041208

[40] Houle J, Meunier S, Coulombe S, Mercerat C, Gaboury I, remblay G, de Montigny F, Cloutier L, Roy B, Auger N, Lavoie B. Peer positive social control and men’s health-promoting behaviors. Am J Mens Health. 2017;11(5):1569–79. 28670962 10.1177/1557988317711605 PMC5675192

[41] Pruckner GJ, Schober T, Zocher K. The company you keep: health behavior among work peers. Eur J Health Econ. 2020;21:251–9. 31664627 10.1007/s10198-019-01124-4 PMC7072047

[42] Kanchan S, Gaidhane A. Social media role and its impact on public health: a narrative review. Cureus. 2023;15(1):e33737. 36793805 10.7759/cureus.33737 PMC9925030

[43] Hoffman SJ, Mansoor Y, Natt N, Sritharan L, Belluz J, Caulfield T, Freedhoff Y, Lavis JN, Sharma AM. Celebrities’ impact on health-related knowledge, attitudes, behaviors, and status outcomes: protocol for a systematic review, meta-analysis, and meta-regression analysis. Syst Rev. 2017;6:13. 28109320 10.1186/s13643-016-0395-1 PMC5251292

[44] Tisler D, Norden L. Securing the 2024 election. Brennan center for justice. 2023. https://www.brennancenter.org/our-work/policy-solutions/securing-2024-election. Accessed 24 June 2025.

[45] Clapman A. How states can prevent election subversion in 2024 and beyond. Brennan Center for Justice. 2023. https://www.brennancenter.org/our-work/research-reports/how-states-can-prevent-election-subversion-2024-and-beyond. Accessed 24 June 2025.

[46] Carey T, Roskam K, Horwitz J, Johns Hopkins Bloomberg School of Public Health. Defending democracy: addressing the dangers of armed insurrection. Center for Gun Violence Solutions. 2023. https://publichealth.jhu.edu/2023/preventing-armed-insurrection-firearms-in-political-spaces-threaten-public-health-safety-and-democracy. Accessed 24 June 2025.

[47] Morales-Doyle S, Sanders R, Anderman A, Ojeda J. Guns and voting: how to protect elections after Bruen. Brennan Center for Justice, New York University School of Law; Giffords Law Center to Prevent Gun Violence. 2023. https://www.brennancenter.org/our-work/policy-solutions/guns-and-voting. Accessed 24 June 2025.

[48] Department of Homeland Security. If you see something, say something. [Website] 2024. https://www.dhs.gov/see-something-say-something. Accessed 24 June 2025.

[49] National Counterterrorism Center, Office of the Director of National Intelligence. U.S. violent extremist mobilization indicators 2021. https://www.dni.gov/files/NCTC/documents/news_documents/Mobilization_Indicators_Booklet_2021.pdf. Accessed 24 June 2025.

