# Supplementary material for: Public opinion on civil war in the USA as of mid-2024: findings from a nationally representative survey
Source: Inj Epidemiol. 2025 Jul 3;12:36. doi: 10.1186/s40621-025-00594-w (PMC12225159; doi:10.1186/s40621-025-00594-w)
Supplement: Supplementary file 1 — Additional file 1: Supplement: Public Opinion on Civil War in the USA as of Mid-2024: Findings from a Nationally Representative Survey Description of data: Questions that supplied data for this analysis, supplemental methods text, 1 figure, and 13 tables [file 40621_2025_594_MOESM1_ESM.pdf]

Supplement

Public Opinion on Civil War in the USA as of Mid-2024:

Findings from a Nationally Representative Survey

Garen J. Wintemute, MD, MPH; Yueju Li, MA; Mona A. Wright, MPH; Andrew Crawford, PhD; Elizabeth A. Tomsich, PhD

This supplement has been provided by the authors to give readers additional information about the work.

Page	Title
2	Questions that supplied data for this study
12	Additional methods text
17	Additional results text
20	References
21	Figure S1. Flowchart of survey assignment and completion n for Waves 1-3 in 2022-2024
22	Table S1. Sociodemographic characteristics (unweighted) of respondents and non-respondents in the 2022-2024 political violence surveys
24	Table S2. Sociodemographic characteristics of respondents for this study
26	Table S3. Association between political party affiliation and views on civil war in the United States
29	Table S4. Association between self-reported MAGA status and views on civil war in the United States
31	Table S5. Association between political ideology and views on civil war in the United States
34	Table S6. Association between beliefs on democracy and authoritarianism and views on civil war in the United States
37	Table S7. Association between beliefs about race and ethnicity and views on civil war in the United States
39	Table S8. Association between beliefs about violence to effect social change and views on civil war in the United States
41	Table S9. Association between approval of extreme right-wing organizations and social movements and views on civil war in the United States
43	Table S10. Association between firearm ownership status and views on civil war in the United States
45	Table S11. Association between types(s) of firearm owned and views on civil war in the United States
47	Table S12. Association between recency of firearm purchase and views on civil war in the United States
49	Table S13. Association between frequency of firearm carrying and views on civil war in the United States

QUESTIONS THAT SUPPLIED DATA FOR THIS STUDY

Response options are presented here in order from negative to positive (e.g., "not important" to "extremely important"). Respondents were randomized 1:1 to receive responses in that order or the reverse.

In the list below, questions or items that were repeated or adapted from prior surveys by other investigators contain citations to those surveys.

Civil war (Wave 3, 2024)

Q: How much do you agree or disagree with each of the following statements?

a. In the next few years, there will be civil war in the United States.[1]

b. The United States needs a civil war to set things right.

1. Do not agree
2. Somewhat agree
3. Strongly agree
4. Very strongly agree

Earlier we asked if you agreed that there would be civil war in the United States in the next few years. The next few items are about what might happen if civil war did occur.

Q: Suppose a civil war occurred and took the form of a RIGHT-wing anti-government insurgency. Which side would you most likely support? Please choose one of the following.

a. The insurgency

b. The government

c. Neither side

Q: Suppose a civil war occurred and took the form of a LEFT-wing anti-government insurgency. Which side would you most likely support? Please choose one of the following.

a. The insurgency

b. The government

c. Neither side

Q: If a civil war did occur, how likely would you be to do each of the following?

a. Leave the United States

b. “Sit it out”: stay in the United States, without participating in the conflict

c. Participate in a non-combat role (examples: providing food and supplies, working as a mechanic or medic)

d. Participate as a combatant (someone who is fighting)

e Kill a combatant from the opposing side

f. Kill a non-combatant from the opposing side

1. Not likely
2. Somewhat likely
3. Very likely
4. Extremely likely

(Asked if the response to “participate as a combatant” was “not likely.”)

Q: You said that it was “not likely” that you would participate as a combatant (someone who is fighting). Which of the following, if any, would make you “very likely” or “extremely likely” to participate in the fighting? Please indicate “yes” or “no” for each one.

a. Members of your family urge you to be a combatant.

b. Your friends urge you to be a combatant.

c. A religious leader you respect urges people like you to be combatants.

d. An elected official or other public figure you respect urges people like you to be combatants.

e. A news or social media source you respect urges people like you to be combatants.

1. No
2. Yes

(Asked if the response to “participate as a combatant” was “very likely” or “extremely likely.”)

Q: You said that it was [“very likely” OR “extremely likely”] that you would participate as a combatant (someone who is fighting). Which of the following, if any, would make you “not likely” to participate in the fighting? Please indicate “yes” or “no” for each one.

a. Members of your family urge you not to be a combatant.

b. Your friends urge you not to be a combatant.

c. A religious leader you respect urges people like you not to be combatants.

d. An elected official or other public figure you respect urges people like you not to be combatants.

e. A news or social media source you respect urges people like you not to be combatants.

1. No

2. Yes

Party affiliation and MAGA status (Wave 3, 2024)

Q: Generally speaking, do you think of yourself as...*Select one answer only.*

1. Republican

2. Democrat

3. Independent

4. Something else

(Asked if Republican)

Q: Would you call yourself a...*Select one answer only.*

1. Strong Republican

2. Not very strong Republican

(Asked if Democrat)

Q: Would you call yourself a...*Select one answer only.*

1. Strong Democrat

2. Not very strong Democrat

(Asked if Independent or Something else)

Q: Do you think of yourself as closer to the...*Select one answer only.*

1. Republican Party

2. Democratic Party

3. Do not lean either way

(Asked if Republicans OR Leans Republican)

Q: Do you think of yourself as a MAGA Republican?

1. No

2. Yes

(Asked if Not MAGA Republican OR Democrat / Leans Democrat)

Q: Do you think of yourself as a supporter of the MAGA movement?

1. No

2. Yes

Democracy and authoritarianism (Wave 3, 2024)

Q. How much do you agree or disagree with the following statements about democracy in the United States?

c. Having a strong leader for America is more important than having a democracy.

e. We should suspend Congress for a few years so a strong leader can clean up the mess made by politicians in Washington.[2]

1. Do not agree
2. Somewhat agree
3. Strongly agree
4. Very strongly agree

Race and ethnicity (Wave 2, 2023)

Q: How much do you agree or disagree with each of the following statements about people in America today?

a. White people benefit from advantages in society that Black people do not have.[1] (Reverse coded)

b. Discrimination against whites is as big a problem as discrimination against Blacks and other minorities.[4]

d. In America, native-born white people are being replaced by immigrants.

e. Having more Black Americans, Latinos, and Asian Americans is good for the country.[5] (Reverse coded)

1. Do not agree
2. Somewhat agree
3. Strongly agree
4. Very strongly agree

Violence to effect social change (Wave 3, 2024)

Q: How much do you agree or disagree with the following statements about democracy in the United States?

d. If elected leaders will not protect American democracy, the people must do it themselves, even if it requires taking violent actions.[3]

1. Do not agree
2. Somewhat agree
3. Strongly agree
4. Very strongly agree

Q: How much do you agree or disagree with each of the following statements about people in America today?

c. Our American way of life is disappearing so fast that we may have to use force to save it.[3]

1. Do not agree
2. Somewhat agree
3. Strongly agree
4. Very strongly agree

Q: People have many different views about American society. How much do you agree or disagree with each of the following?

c. Because things have gotten so far off track, true American patriots may have to resort to violence in order to save our country.[6]

1. Do not agree
2. Somewhat agree
3. Strongly agree
4. Very strongly agree

Approval of specified organizations and movements (Wave 1, 2022)

Q: How much do you approve or disapprove of these named groups and organizations?

Proud Boys

Oath Keepers

Three Percenters

QAnon

1. Do not approve
2. Somewhat approve
3. Strongly approve
4. Very strongly approve
5. I don’t know enough about this group or organization to rate it
6. I have never heard of this group or organization

Q: How much do you approve or disapprove of these political or social movements?

The militia movement

The white supremacy movement

The Christian nationalist movement

The boogaloo movement

1. Do not approve
2. Somewhat approve

3. Strongly approve
4. Very strongly approve
5. I don't know enough about this group or organization to rate it [ANCHOR]
6. I have never heard of this group or organization [ANCHOR]

Firearm ownership (Wave 1, 2022)

Ownership Status

Q: Do you happen to keep any guns in your home or garage?

1. Yes
2. No

(Asked if the response to the prior question was "yes.")

Q: Do any of these guns personally belong to you?

1. Yes
2. No

Type(s) of Firearm Owned

(Asked if the response to the prior question was "yes." Presented with firearm types in rows and responses in columns. Respondents gave separate answers for each firearm type.)

Q: Do you personally own any of the following types of guns?

a. Handguns

b. Rifles

c. Shotguns

d. Other types of guns

1. Yes
2. No

(Asked if the response to "rifles" in the prior question was "yes.")

Q: Do you own any rifles of the type sometimes called tactical rifles, or modern sporting rifles, or assault rifles, such as an AR-15, an AK-47, or an SKS?

1. Yes

2. No

Recency of Purchase

(Asked if the response to the personal ownership question was "yes.")

Q: Did you buy any guns this year, in 2022?

1. Yes

2. No

Q: Did you buy any guns in 2021?

1. Yes

2. No

Q: Did you buy any guns in 2020?

1. Yes

2. No

Q: When you bought guns in (insert earliest year from the series above) did you already own any guns?

1. Yes

2. No

Carrying Behavior

(Asked of all respondents)

Q: In the last year, have you carried a loaded gun (handgun, rifle, or shotgun) on your person when you were out in public? Do not include hunting, time at a shooting range, or similar activities.

1. Yes

2. No

(Asked if the response to the prior question was "yes.")

Q: In the last year, and on days when you were out in public, how often have you carried a loaded gun on your person? Again, do not include hunting, time at a shooting range, or similar activities.

1. Not often at all

2. Less than half the time

3. About half the time

4. More than half the time

5. All (or nearly all) the time

Political violence (Wave 3, 2024)

Now we have a few questions about the use of force or violence. "Force or violence" means physical force strong enough that it could cause pain or injury to a person. A reminder: your responses will be kept confidential and anonymous.

Asked of respondents who considered force or violence to be at least somewhat justified, either in general or to advance at least 1 of a list of specified political objectives.

Q: In a situation where you think force or violence is justified to advance an important political objective, how willing would you personally be to use force or violence in each of these ways?

a. To damage property

b. To threaten or intimidate a person

d. To kill a person

1. Not willing

2. Somewhat willing

3. Very willing

4. Completely willing

(Asked of all respondents.)

Q: Thinking now about the future and all the changes it might bring, how likely is it that you will use a gun in any of the following ways in the next few years—in a situation where you think force or violence is justified to advance an important political objective?

a. I will be armed with a gun.

b. I will carry a gun openly, so that people know I am armed.

d. I will shoot someone with a gun.

1. Not likely

2. Somewhat likely

3. Very likely

4. Extremely likely

Question from the KnowledgePanel profile questionnaire that supplied data for this study

Political ideology

Q: In general, do you think of yourself as...

1. Extremely liberal

2. Liberal

3. Slightly liberal

4. Moderate/middle of the road

5. Slightly conservative

6. Conservative

7. Extremely conservative

ADDITIONAL METHODS TEXT

Construction of Measures

Party affiliation

Respondents were first asked, “Generally speaking, do you think of yourself as...” with response options Republican, Democrat, independent, or something else. Those who responded Republican or Democrat were asked, “Would you call yourself a...” with response options Strong Republican/Democrat and not very strong Republican/Democrat. Those who responded to the initial item with independent or something else, were asked, “Do you think of yourself as closer to the...” with response options Republican Party, Democratic Party, and do not lean either way.

MAGA status

Respondents who identified as Republican, or as independent or something else but closer to the Republican Party than to the Democratic Party, were asked, “Do you think of yourself as a MAGA Republican?” All who responded “no” to that question and all other respondents were asked, “Do you think of yourself as a supporter of the MAGA movement?”

Political ideology

Political ideology was reported to Ipsos by panel members in responding to the question, “In general, do you think of yourself as…” with 7 response options ranging from

“extremely liberal” to “extremely conservative” and with “moderate/middle of the road" as a midpoint.

Beliefs about race and ethnicity, beliefs about violence to effect social change, approval of extreme right-wing organizations and movements

Individual item responses these exposures were coded ordinally (e.g., do not agree = 0, somewhat agree = 1, strongly agree = 2, very strongly agree =3) and summed for each respondent for each of the 3 exposures. Summed scores were normalized to a range from 0 to 1, with 0 and 1 representing the minimum and maximum theoretically possible scores, respectively. Respondents’ normalized scores were then categorized according to their position on that range (e.g., strong agreement, normalized score> 0.66… and ≤ 1; moderate agreement, normalized score> 0.33… and ≤ 0.66; weak agreement, normalized score> 0 and ≤ 0.33…; non-agreement, normalized score = 0). Modified procedures allowed for inclusion of respondents with missing values for up to half the items for any exposure (Table 1 and Supplement).
For each exposure, we calculated Cronbach’s α using individual item scores to assess the internal consistency of those items, with bootstrapped 95% confidence intervals (CI) based on 500 samples.

Firearm ownership and use

Respondents were categorized as personal firearm owners, nonowners with firearms at home, and nonowners without firearms at home. Firearm owners were categorized in 4 groups based on the type(s) of firearm they owned: assault-type rifle owners (owns 1 or more assault-

type rifles; may own firearms of other types), other rifle owners (owns 1 or more rifles that are not-assault type rifles; does not own assault-type rifles; may own firearms of other types), handgun-only owners (owns 1 or more handguns and no firearms of other types), other owners (owns any combination of handguns, shotguns, and firearms of other types; does not own rifles). Firearm owners were also categorized in 2 groups by recency of most recent purchase: 2020 or later, and 2019 or earlier. Finally, owners were categorized in 4 groups based on their frequency of carrying a loaded firearm on their person when out in public (with sporting use excluded): never/not often at all; less than half the time/about half the time/more than half the time; and always/nearly always.

Political Violence

Violence was represented by the phrase “force or violence,” defined in the questionnaire as “physical force strong enough that it could cause pain or injury to a person.” “Force or violence to advance an important political objective that you support” was used in questions about respondents’ support for and willingness to engage in political violence.

Respondents in 2024 who considered political violence to be at least sometimes justified for at least 1 objective were asked about their personal willingness to engage in political violence: by type of violence (to “damage property,” “threaten or intimidate a person,” “injure a person,” “kill a person”) and by target population (examples: “an elected federal or state government official,” “a police officer,” “a person who does not share your religion”).

All respondents were asked about the likelihood of their future use of firearms in a situation where they consider political violence to be justified (examples: "I will be armed with a gun," "I will shoot someone with a gun").

Implementation

To help minimize overestimation of support for political violence, questions regarding political violence were immediately preceded by a question about the justifiability of the use of force or violence in 7 non-political situations. These situations were presented in a fixed order that, in the judgment of the authors, proceeded from more likely to less likely to be seen by respondents as justifying violence: from "in self-defense" to "to get respect." This was done to create an expected response transition from support to nonsupport for violence that respondents would need to reverse to indicate support for political violence.

Statistical analysis

In conducting the analysis of Wave 2 (2023) items pertaining to civil war, we examined the following models for adjusting prevalence differences:

Model 0: unadjusted;

Model 1: adjusted for age (numerical), race and ethnicity (White, Non-Hispanic; Black, Non-Hispanic; Other, Non-Hispanic; Hispanic; 2+ Races, Non-Hispanic), and gender (Male, Female);

Model 2: additionally adjusted for income (Less than $10,000, $10,000 to $24,999, $25,000 to $49,999, $50,000 to $74,999, $75,000 to $99,999, $100,000 to $149,999, $150,000 or

more), education (No high school diploma or GED, High school graduate (high school diploma or the equivalent GED), Some college or Associate's degree, Bachelor's degree, Master’s degree or higher), and Census division (New England, Mid-Atlantic, East-North Central, West-North Central, South Atlantic, East-South Central, West-South Central, Mountain, Pacific);

Model 3: additionally adjusted for rurality (Urban, Rural; derived from Rural-Urban Commuting Codes matched to census tracts (https://www.ers.usda.gov/data-products/rural-urban-commuting-area-codes/).

We used Model 3 for the Wave 2 analysis and retained it for Wave 3 to preserve comparability. Findings from Model 3 appear in the ‘Adjusted prevalence difference” rows in tables. Q-values were also produced using Model 3.

ADDITIONAL RESULTS TEXT

Three items included in this analysis had nonresponse percentages above 3.0%. Items with nonresponse percentages above 3.0% and between 2.0% and 3.0% are listed here, in questionnaire order.

Nonresponse above 3.0%

Q. Do you think of yourself as a supporter of the MAGA movement?

Nonresponse = 3.5 %

Q. People have many different views about society in the United States. How much do you agree or disagree with each of the following? - The government, media, and financial worlds in the U.S. are controlled by a group of Satan-worshipping pedophiles who run a global child sex trafficking operation.

Nonresponse = 3.1%

Q. Suppose a civil war occurred and took the form of a LEFT-wing anti-government insurgency. Which side would you most likely support? Please choose one of the following.

a. The insurgency
b. The government
c. Neither side

Nonresponse = 3.01 %

Nonresponse between 2.0% and 3.0%

Q. How much do you agree or disagree with the following statements about democracy in the United States? - Having a strong leader for America is more important than having a democracy.

Nonresponse = 2.1%

Q. How much do you agree or disagree with each of the following statements?

In the next few years, there will be civil war in the United States.
Nonresponse =2.3%

The United States needs a civil war to set things right.

Nonresponse =2.3%

Q. How likely is it that you will use a gun in any of the following ways in the next few years—in a situation where you think force or violence is justified to advance an important political objective? - I will be armed with a gun

Nonresponse = 2.4%

Q. How likely is it that you will use a gun in any of the following ways in the next few years—in a situation where you think force or violence is justified to advance an important political objective? - I will carry a gun openly, so that people know

Nonresponse = 2.5%

Q. How likely is it that you will use a gun in any of the following ways in the next few years—in a situation where you think force or violence is justified to advance an important political objective? - I will shoot someone with a gun.

Nonresponse = 2.3%

Q. Suppose a civil war occurred and took the form of a RIGHT-wing anti-government insurgency. Which side would you most likely support? Please choose one of the following.

a. The insurgency
b. The government
c. Neither side

Nonresponse =2.9%

Q. If a civil war did occur, how likely would you be to do each of the following?

a. Leave the United States
Nonresponse = 2.3%

b. "Sit it out": stay in the United States, without participating in the conflict
Nonresponse = 2.4%

c. Participate in a non-combat role (examples: providing food and supplies, working as a mechanic or medic)
Nonresponse = 2.7%

d. Participate as a combatant (someone who is fighting)
Nonresponse = 2.6%

e. Kill a combatant from the opposing side
   Nonresponse = 2.7%

f. Kill a non-combatant from the opposing side
   Nonresponse = 2.9%

Q. In general, how willing would you personally be to take action to prevent another person from using force or violence to advance a political objective when you do not think the use of force or violence is justified?

Nonresponse = 2.97%

REFERENCES

1. Zogby. Will the US have another civil war? 2021 Feb 4. https://zogbyanalytics.com/news/997-the-zogby-poll-will-the-us-have-another-civil-war.

2. Democracy Fund Voter Study Group. Guide to the views of the Electorate Research Survey. 2021 December. https://www.voterstudygroup.org/data/voter-survey.

3. Pew Research Center. Deep divisions in Americans' views of nation's racial history – and how to address it. 2021 August. https://www.pewresearch.org/politics/2021/08/12/deep-divisions-in-americans-views-of-nations-racial-history-and-how-to-address-it/.

4. Cox D, Lienesch R, Jones RP. Beyond economics: fears of cultural displacement pushed the white working class to Trump | PRRI/The Atlantic Report. Public Religion Research Institute. 2019 May 17. https://www.prri.org/research/white-working-class-attitudes-economy-trade-immigration-election-donald-trump/.

5. Pew Research Center. Americans see advantages and challenges in country's growing racial and ethnic diversity. 2019 May. https://www.pewresearch.org/social-trends/2019/05/08/americans-see-advantages-and-challenges-in-countrys-growing-racial-and-ethnic-diversity/.

6. IFYC – PRRI Survey on Religion & COVID-19 Vaccine Trust. 2021 March. https://www.prri.org/wp-content/uploads/2021/05/Topline-IFYC-PRRI-Survey-on-Religion-and-COVID-19-Vaccine-Trust-v2_final.pdf.

Figure S1. Flowchart of survey assignment and completion n for Waves 1-3 in 2022-2024

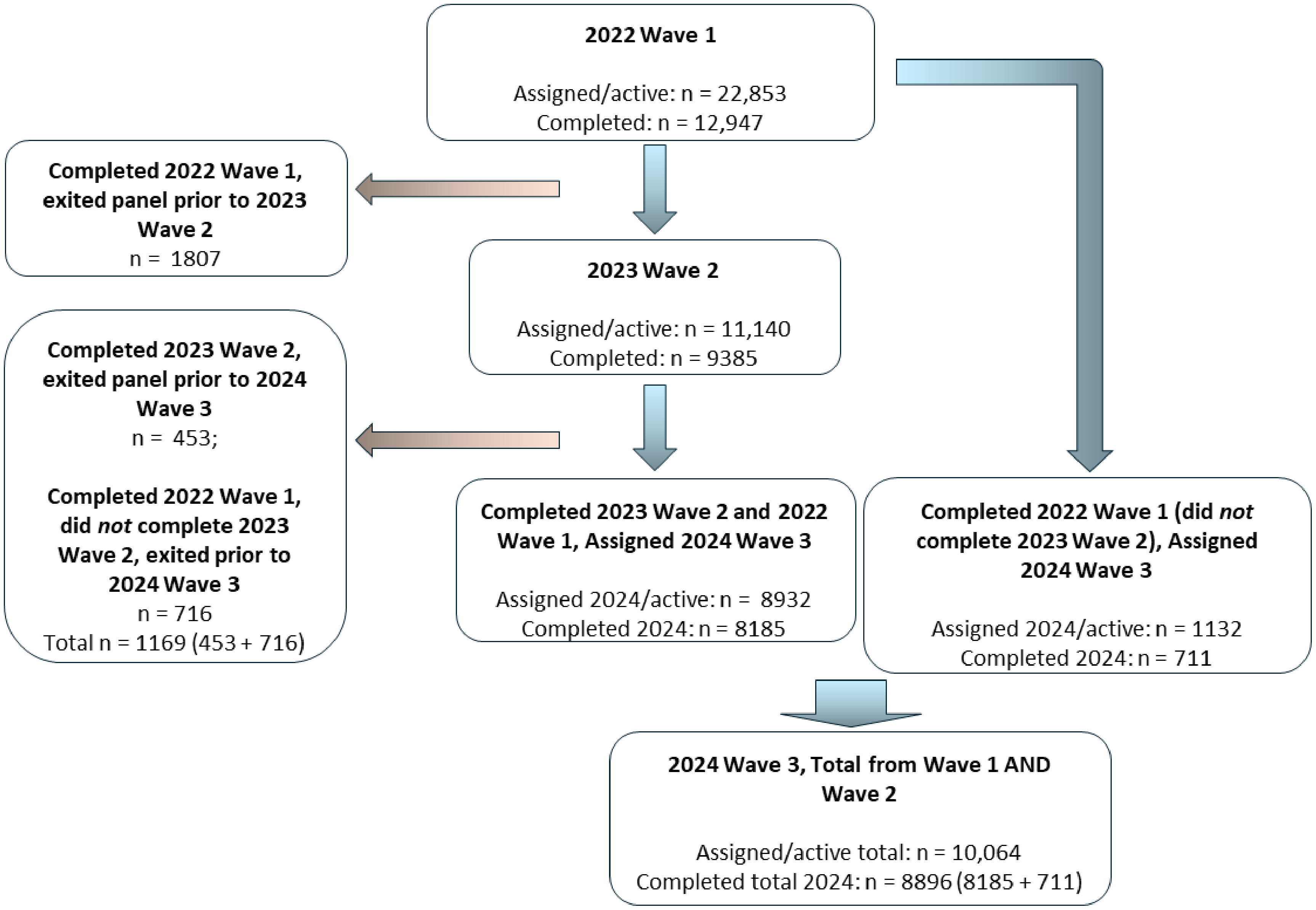

Table S1. Sociodemographic characteristics (unweighted) of respondents and non-respondents in the 2022-2024 political violence surveys

Characteristics	2022 (Wave 1)				Wave 1 respondents who left the panel prior to Wave 2 (n = 1,807)	
	Respondents* (n = 12,947)		Non-respondents (n = 9906)			
	Unweighted n	Unweighted %	Unweighted n	Unweighted %	Unweighted n	Unweighted %
**Age**						
18-24	488	3.8	1121	11.3	86	4.8
25-34	1309	10.1	1575	15.9	210	11.6
35-44	1884	14.6	1969	19.9	326	18
45-54	1847	14.3	1882	19.0	335	18.5
55-64	2794	21.6	1597	16.1	391	21.6
65-74	2952	22.8	1188	12.0	313	17.3
75+	1673	12.9	574	5.8	146	8.1
Non-response	0	0.0	0	0.0	0	0.0
**Gender**						
Male	7158	55.3	4690	47.3	854	47.3
Female	5789	44.7	5216	52.7	953	52.7
Non-response	0	0.0	0	0.0	0	0.0
**Race/Ethnicity**						
Black, non-Hispanic	1097	8.5	1039	12.5	170	9.4
Hispanic, any race	1504	11.6	1561	18.8	237	13.1
White, non-Hispanic	9493	73.3	5030	60.5	1272	70.4
Other, non-Hispanic	499	3.9	370	4.4	77	4.3
2+races, non-Hispanic	354	2.7	318	3.8	51	2.8
Non-response	0	0.0	0	0.0	0	0.0
**Marital status**						
Now married	8074	62.4	5443	54.9	1089	60.3
Widowed	770	5.9	405	4.1	82	4.5
Divorced	1456	11.2	1049	10.6	240	13.3
Separated	193	1.5	223	2.3	34	1.9
Never married	2454	19.0	2786	28.1	362	20
Non-response	0	0.0	0	0.0	0	0.0
**Education**						
No high school diploma or GED	624	4.8	754	7.6	121	6.7
High school graduate (diploma, G	2813	21.7	2410	24.3	452	25
Some college or Associate's degr	3896	30.1	3173	32.0	584	32.3
Bachelor's degree	3133	24.2	2117	21.4	372	20.6
Master's degree or higher	2481	19.2	1452	14.7	278	15.4
Non-response	0	0.0	0	0.0	0	0.0
**Household Income**						
Less than $10,000	371	2.9	489	4.9	72	4
$10,000 to $24,999	1078	8.3	935	9.4	189	10.5
$25,000 to $49,999	2232	17.2	1829	18.5	318	17.6
$50,000 to $74,999	2236	17.3	1709	17.3	313	17.3
$75,000 to $99,999	1999	15.4	1458	14.7	236	13.1
$100,000 to $149,999	2410	18.6	1745	17.6	336	18.6
$150,000 or more	2621	20.2	1741	17.6	343	19
Non-response	0	0.0	0	0.0	0	0.0
**Employment**						
Working full-time	5645	43.6	5252	53.0	889	49.2
Working part-time	1620	12.5	1562	15.8	258	14.3
Not working	5682	43.9	3092	31.2	660	36.5
Non-response	0	0.0	0	0.0	0	0.0
**Census division**						
New England	509	3.9	371	3.7	73	4
Mid-Atlantic	1407	10.9	1068	10.8	191	10.6
East-North Central	1878	14.5	1356	13.7	262	14.5
West-North Central	952	7.4	729	7.4	137	7.6
South Atlantic	2538	19.6	1978	20.0	326	18
East-South Central	737	5.7	701	7.1	117	6.5
West-South Central	1371	10.6	1253	12.6	207	11.5
Mountain	1125	8.7	695	7.0	156	8.6
Pacific	2430	18.8	1755	17.7	338	18.7
Non-response	0	0.0	0	0.0	0	0.0

Table S1, continued.

Characteristics	2023 (Wave 2)				Wave 2 respondents who left the panel prior to Wave 3 (n = 453)		2024 (Wave 3)			
	Respondents* (n = 9,385)		Non-respondents (n = 1,755)				Respondents* (n=8896)		Non-respondents (n = 1168)	
	Unweighted n	Unweighted %	Unweighted n	Unweighted %	Unweighted n	Unweighted %	Unweighted n	Unweighted %	Unweighted n	Unweighted %
**Age**										
18-24	310	3.3	92	5.2	11	2.4	176	2.0	64	5.5
25-34	856	9.1	243	13.8	54	11.9	753	8.5	139	11.9
35-44	1252	13.3	306	17.4	50	11.0	1094	12.3	192	16.4
45-54	1255	13.4	257	14.6	63	13.9	1150	12.9	169	14.5
55-64	2043	21.8	360	20.5	100	22.1	1827	20.5	232	19.9
65-74	2342	25.0	297	16.9	96	21.2	2249	25.3	239	20.5
75+	1327	14.1	200	11.4	79	17.4	1647	18.5	133	11.4
Non-response	0	0.0	0	0.0	0	0.0	0	0.0	0	0.0
**Gender**										
Male	5437	57.9	867	49.4	261	57.6	5147	57.9	580	49.7
Female	3948	42.1	888	50.6	192	42.4	3749	42.1	588	50.3
Non-response	0	0.0	0	0.0	0	0.0	0	0.0	0	0.0
**Race/Ethnicity**										
Black, non-Hispanic	749	8.0	178	10.1	27	6.0	702	7.9	128	11.0
Hispanic, any race	1016	10.8	251	14.3	49	10.8	959	10.8	181	15.5
White, non-Hispanic	7014	74.7	1207	68.8	351	77.5	6673	75.0	778	66.6
Other, non-Hispanic	346	3.7	76	4.3	18	4.0	316	3.6	49	4.2
2+races, non-Hispanic	260	2.8	43	2.5	8	1.8	246	2.8	32	2.7
Non-response	0	0.0	0	0.0	0	0.0	0	0.0	0	0.0
**Marital status**										
Now married	5961	63.5	1024	58.3	273	60.3	5655	63.6	681	58.3
Widowed	582	6.2	106	6	36	7.9	630	7.1	83	7.1
Divorced	1010	10.8	206	11.7	61	13.5	979	11.0	117	10
Separated	122	1.3	37	2.1	5	1.1	127	1.4	17	1.5
Never married	1710	18.2	382	21.8	78	17.2	1505	16.9	270	23.1
Non-response	0	0.0	0	0.0	0	0.0	0	0.0	0	0.0
**Education**										
No high school diploma or GED	416	4.4	87	5	17	3.8	330	3.7	66	5.7
High school graduate (diploma, G	2002	21.3	359	20.5	121	26.7	1784	20.1	253	21.7
Some college or Associate's degr	2773	29.5	539	30.7	147	32.5	2691	30.2	354	30.3
Bachelor's degree	2337	24.9	424	24.2	85	18.8	2257	25.4	279	23.9
Master's degree or higher	1857	19.8	346	19.7	83	18.3	1834	20.6	216	18.5
Non-response	0	0.0	0	0.0	0	0.0	0	0.0	0	0.0
**Household Income**										
Less than $10,000	233	2.5	66	3.8	13	2.9	265	3	40	3.4
$10,000 to $24,999	727	7.7	162	9.2	52	11.5	609	6.8	132	11.3
$25,000 to $49,999	1617	17.2	297	16.9	89	19.6	1446	16.3	220	18.8
$50,000 to $74,999	1631	17.4	292	16.6	76	16.8	1424	16	220	18.8
$75,000 to $99,999	1499	16.0	264	15	61	13.5	1367	15.4	160	13.7
$100,000 to $149,999	1734	18.5	340	19.4	72	15.9	1799	20.2	206	17.6
$150,000 or more	1944	20.7	334	19	90	19.9	1986	22.3	190	16.3
Non-response	0	0.0	0	0.0	0	0.0	0	0.0	0	0
**Employment**										
Working full-time	3869	41.2	887	50.5	181	40	3593	40.4	525	44.9
Working part-time	1133	12.1	229	13	59	13	1038	11.7	160	13.7
Not working	4383	46.7	639	36.4	213	47	4265	47.9	483	41.4
Non-response	0	0.0	0	0.0	0	0.0	0	0.0	0	0.0
**Census division**										
New England	374	4.0	62	3.5	13	2.9	362	4.1	42	3.6
Mid-Atlantic	1001	10.7	215	12.3	46	10.2	960	10.8	148	12.7
East-North Central	1370	14.6	246	14	52	11.5	1306	14.7	178	15.2
West-North Central	676	7.2	139	7.9	44	9.7	647	7.3	72	6.2
South Atlantic	1881	20.0	331	18.9	105	23.2	1754	19.7	233	19.9
East-South Central	538	5.7	82	4.7	23	5.1	514	5.8	63	5.4
West-South Central	965	10.3	199	11.3	47	10.4	902	10.1	123	10.5
Mountain	825	8.8	144	8.2	37	8.2	796	8.9	87	7.4
Pacific	1755	18.7	337	19.2	86	19.0	1655	18.6	222	19.0
Non-response	0	0.0	0	0.0	0	0.0	0	0.0	0	0.0

* Most values are as of 2022; census division values were updated for 2024; other demographics were not re-asked.

Mean [SD] ages were as follows: Wave 1 responders, 55.7 (16.7); Wave 1 non-responders, 46.9 (16.8); Wave 1 respondents who left the panel prior to Wave 2, 52.17 (16.2); Wave 2 responders, 56.99 (16.5); Wave 2 non-responders, 52.47 (17.5); Wave 3 responders, 56.8 (16.5); Wave 3 non-responders, 53.8 (17.5); Wave 2 respondents who left the panel prior to Wave 3, 57.6 (17.4).

Table S2. Sociodemographic characteristics of respondents for this study

Characteristic	2024 Respondents (n= 8185)	
	Unweighted n	Weighted % (95% CI)
**Age**		
18-24	159	6.6 (5.5, 7.6)
25-34	673	17.0 (15.7, 18.3)
35-44	979	16.7 (15.6, 17.9)
45-54	1053	14.8 (13.8, 15.8)
55-64	1664	18.4 (17.3, 19.4)
65-74	2113	15.5 (14.7, 16.3)
75+	1544	11.1 (10.3, 11.8)
Non-response	0	
**Gender**		
Female	3323	50.3 (48.8, 51.7)
Male	4708	47.6 (46.1, 49.1)
Transgender	39	0.4 (0.2, 0.7)
Non-binary	51	0.7 (0.5, 1)
Other	19	0.3 (0.1, 0.5)
Non-response	45	0.6 (0.3, 0.9)
**Race/Ethnicity**		
American Indian or Alaska Native, Non-Hispani	39	1.0 (0.6, 1.4)
Asian American or Pacific Islander, non-Hispan	239	5.3 (4.5, 6.2)
Black, Non-Hispanic	651	11.9 (10.8, 13.0)
Hispanic, any race	847	16.5 (15.2, 17.8)
White, Non-Hispanic	6159	63.3 (61.8, 64.9)
Some other race, Non-Hispanic	18	0.1 (0.1, 0.2)
2+ Races, Non-Hispanic	232	1.8 (1.4, 2.2)
Non-response	0	
**Marital status**		
Now married	5220	57.3 (55.8, 58.8)
Widowed	587	4.8 (4.3, 5.3)
Divorced	885	8.4 (7.7, 9.0)
Separated	118	1.8 (1.4, 2.3)
Never married	1375	27.8 (26.3, 29.3)
Non-response	0	

Table S2, continued.

Characteristic	2024 Respondents (n= 8185)	
	Unweighted n	Weighted % (95% CI)
**Education**		
No high school diploma or GED	305	7.4 (6.4, 8.4)
High school graduate (diploma, GED)	1657	27.3 (25.9, 28.7)
Some college or Associate's degree	2461	28.4 (27.1, 29.7)
Bachelor's degree	2090	21.2 (20.0, 22.3)
Master's degree or higher	1672	15.8 (14.9, 16.7)
Non-response	0	
**Household Income**		
Less than $10,000	231	4.6 (3.9, 5.4)
$10,000 to $24,999	572	8.0 (7.1, 8.9)
$25,000 to $49,999	1336	17.1 (16.0, 18.3)
$50,000 to $74,999	1315	15.3 (14.3, 16.4)
$75,000 to $99,999	1269	13.5 (12.5, 14.4)
$100,000 to $149,999	1642	18.4 (17.3, 19.4)
$150,000 or more	1820	23.1 (21.9, 24.3)
Non-response	0	
**Employment**		
Working - as a paid employee	3734	52.5 (51.0, 53.9)
Working - self-employed	608	7.1 (6.3, 7.8)
Not working - on temporary layoff from a job	32	0.5 (0.3, 0.8)
Not working - looking for work	229	4.8 (4.1, 5.6)
Not working - retired	2978	22.2 (21.2, 23.2)
Not working - disabled	238	4.5 (3.8, 5.2)
Not working - other	366	8.4 (7.4, 9.4)
Non-response	0	
**Census division**		
New England	335	4.6 (3.9, 5.2)
Mid-Atlantic	873	12.4 (11.4, 13.4)
East-North Central	1202	14.4 (13.4, 15.4)
West-North Central	590	6.3 (5.7, 7.0)
South Atlantic	1631	20.8 (19.6, 22.0)
East-South Central	483	6.1 (5.3, 6.8)
West-South Central	824	11.5 (10.5, 12.5)
Mountain	729	7.7 (6.9, 8.5)
Pacific	1518	16.2 (15.2, 17.3)
Non-response	0	

Table S3. Association between political party affiliation and views on civil war in the United States

Query and Response	Party Affiliation							
	Strong Democrat		Not Very Strong Democrat		Leans Democrat		Independent/Other	
	Unweighted n	Weighted % (95% CI)	Unweighted n	Weighted % (95% CI)	Unweighted n	Weighted % (95% CI)	Unweighted n	Weighted % (95% CI)
How much do you agree or disagree with each of the following statements?†								
In the next few years, there will be civil war in the United States.								
Do not agree	998	65.4 (62.0, 68.7)	621	63.8 (59.6, 67.9)	718	66.3 (62.5, 70.2)	740	59.2 (55.4, 63.0)
Somewhat agree	401	28 (24.9, 31.2)	278	28.4 (24.5, 32.2)	311	30.1 (26.4, 33.9)	320	27.1 (23.7, 30.5)
Strongly or very strongly agree	61	5.5 (3.5, 7.5)	44	6.2 (3.8, 8.5)	28	3.5 (1.9, 5.1)	93	9.4 (7.1, 11.8)
Adjusted prevalence difference (95% CI; q-value)*	-2.8 (-5.8, 0.2; 0.24)		-2.6 (-6.0, 0.8; 0.35)		-4.0 (-6.9, -1.0; 0.06)		Referent	
The United States needs a civil war to set things right.								
Do not agree	1403	92.4 (90.1, 94.7)	872	88.6 (85.5, 91.7)	1008	92.8 (90.2, 95.3)	998	81.0 (77.9, 84.2)
Somewhat agree	36	4.0 (2.4, 5.5)	52	6.3 (4.0, 8.7)	45	6.6 (4.1, 9.0)	107	9.2 (6.8, 11.6)
Strongly or very strongly agree	28	3.3 (1.7, 5.0)	17	3.0 (1.4, 4.6)	5	0.7 (0.0, 1.5)	48	5.2 (3.5, 7.0)
Adjusted prevalence difference (95% CI; q-value)*	-1.3 (-3.7, 1.0; 0.56)		-1.9 (-4.4, 0.6; 0.35)		-3.7 (-5.7, -1.7; 0.01)		Referent	
Suppose a civil war occurred and took the form of a RIGHT-wing anti-government insurgency. Which side would you most likely support?								
Neither side	438	37.6 (34.2, 41.1)	451	56.6 (52.5, 60.7)	357	39.5 (35.4, 43.5)	787	72.5 (69.3, 75.7)
The government	984	58.3 (54.8, 61.8)	469	38.9 (34.9, 42.8)	672	57.4 (53.4, 61.5)	315	19.9 (17.2, 22.7)
The insurgency	25	2.5 (1.2, 3.7)	22	3.5 (1.7, 5.2)	22	2.3 (1.1, 3.5)	52	3.3 (2.1, 4.6)
Adjusted prevalence difference (95% CI; q-value)†	0.3 (-1.8, 2.4; 0.92)		0.9 (-1.5, 3.2; 0.78)		-0.4 (-2.3, 1.6; 0.92)		Referent	
Suppose a civil war occurred and took the form of a LEFT-wing anti-government insurgency. Which side would you most likely support?								
Neither side	647	50.6 (47.2, 54.1)	520	62.3 (58.3, 66.3)	461	49.0 (44.9, 53.0)	791	72.8 (69.6, 75.9)
The government	639	34.5 (31.4, 37.6)	366	29.5 (25.9, 33.1)	487	39.2 (35.4, 43.1)	319	19.8 (17.1, 22.5)
The insurgency	159	12.9 (10.4, 15.5)	52	6.9 (4.7, 9.1)	99	10.5 (7.8, 13.1)	48	3.4 (2.2, 4.6)
Adjusted prevalence difference (95% CI; q-value)†	10.8 (7.9, 13.8; < 0.001)		4.3 (1.7, 6.8; 0.01)		6.2 (3.2, 9.2; < 0.001)		Referent	
If a civil war did occur, how likely would you be to do each of the following?								
Leave the United States								
Not likely	815	51.6 (48.1, 55.0)	535	51.4 (47.1, 55.6)	547	47.6 (43.6, 51.6)	749	57.7 (53.9, 61.5)
Somewhat likely	430	29.9 (26.7, 33.1)	281	32.0 (28.0, 36.0)	340	33.2 (29.4, 37.1)	282	26.2 (22.7, 29.7)
Very or extremely likely	212	17.5 (14.7, 20.3)	129	15.9 (12.7, 19.2)	166	18.2 (15.1, 21.4)	129	11.8 (9.4, 14.2)
Adjusted prevalence difference (95% CI; q-value)‡	5.2 (1.3, 9.0; 0.06)		2.1 (-2.0, 6.2; 0.63)		6.1 (2.0, 10.2; 0.03)		Referent	
"Sit it out": stay in the United States, without participating in the conflict								
Not likely	201	14.6 (12.1, 17.0)	125	14.4 (11.3, 17.5)	131	13.8 (10.7, 16.8)	177	17.7 (14.6, 20.9)
Somewhat likely	537	35.7 (32.4, 39.0)	344	36.1 (32.0, 40.2)	400	34.8 (31.0, 38.5)	391	31.5 (27.9, 35.0)
Very or extremely likely	719	48.6 (45.2, 52.1)	474	48.7 (44.4, 52.9)	521	50.4 (46.4, 54.4)	585	46.7 (42.9, 50.5)
Adjusted prevalence difference (95% CI; q-value)‡	-1.0 (-6.3, 4.3; 0.92)		0.4 (-5.4, 6.2; 0.95)		1.3 (-4.3, 7.0; 0.88)		Referent	
Participate in a non-combat role (examples: providing food and supplies, working as a mechanic or medic)								
Not likely	487	37.5 (34.1, 41.0)	364	43.1 (38.8, 47.3)	375	37.0 (33.1, 40.8)	488	44.3 (40.5, 48.2)
Somewhat likely	580	37 (33.8, 40.3)	414	39.4 (35.3, 43.5)	439	39.6 (35.7, 43.5)	418	31.8 (28.3, 35.3)
Very or extremely likely	387	24 (21.2, 26.8)	164	16.5 (13.4, 19.7)	237	22.3 (19.0, 25.6)	242	18.9 (16.0, 21.8)
Adjusted prevalence difference (95% CI; q-value)‡	4.8 (0.6, 9.1; 0.13)		-2.9 (-7.3, 1.5; 0.47)		2.7 (-1.8, 7.3; 0.52)		Referent	
Participate as a combatant (someone who is fighting)								
Not likely	1307	88.9 (86.6, 91.2)	846	86.9 (83.8, 90.1)	939	88.8 (86.1, 91.5)	993	84.7 (82.0, 87.4)
Somewhat likely	107	6.7 (4.9, 8.4)	80	10.0 (7.1, 12.8)	90	7.9 (5.6, 10.1)	111	6.9 (5.1, 8.6)
Very or extremely likely	39	3.0 (1.7, 4.4)	19	2.4 (0.9, 3.8)	24	2.4 (1.1, 3.7)	48	4.0 (2.4, 5.5)
Adjusted prevalence difference (95% CI; q-value)‡	-0.1 (-2.0, 1.7; 0.95)		-1.1 (-3.1, 0.9; 0.59)		-0.4 (-2.4, 1.6; 0.91)		Referent	
Kill a combatant from the opposing side								
Not likely	1338	91.2 (89.2, 93.2)	852	89.4 (86.7, 92.1)	957	91.8 (89.5, 94.0)	1020	86.3 (83.8, 88.9)
Somewhat likely	76	4.6 (3.2, 6.0)	69	6.8 (4.7, 9.0)	71	5.1 (3.6, 6.7)	87	5.7 (4.1, 7.2)
Very or extremely likely	39	2.9 (1.6, 4.2)	23	2.7 (1.2, 4.2)	22	2.1 (0.7, 3.5)	43	3.6 (2.2, 4.9)
Adjusted prevalence difference (95% CI; q-value)‡	-0.2 (-2.1, 1.6; 0.92)		-0.7 (-2.8, 1.3; 0.79)		-0.9 (-2.9, 1.1; 0.67)		Referent	
Kill a non-combatant from the opposing side								
Not likely	1414	95 (93.4, 96.7)	909	94.3 (92.2, 96.5)	1030	95.3 (93.0, 97.7)	1099	90.0 (87.6, 92.4)
Somewhat likely	19	1.9 (0.7, 3.1)	27	3.9 (1.9, 5.8)	15	3.0 (1.0, 5.0)	31	3.3 (1.9, 4.8)
Very or extremely likely	21	1.7 (0.8, 2.7)	9	1.1 (0.3, 1.9)	4	0.2 (0.0, 0.5)	20	2.0 (0.9, 3.1)
Adjusted prevalence difference (95% CI; q-value)‡	-0.3 (-1.9, 1.2; 0.89)		-1.1 (-2.5, 0.3; 0.35)		-1.4 (-2.5, -0.2; 0.11)		Referent	

Table S3, continued.

Query and Response	Party Affiliation					
	Leans Republican		Not Very Strong Republican		Strong Republican	
	Unweighted n	Weighted % (95% CI)	Unweighted n	Weighted % (95% CI)	Unweighted n	Weighted % (95% CI)
How much do you agree or disagree with each of the following statements?†						
In the next few years, there will be civil war in the United States.						
Do not agree	617	62.2 (58.0, 66.5)	703	65.4 (61.4, 69.4)	889	54.8 (51.4, 58.3)
Somewhat agree	277	29.7 (25.7, 33.7)	284	29.5 (25.7, 33.3)	470	33.4 (30.1, 36.8)
Strongly or very strongly agree	49	6.6 (4.2, 9.0)	28	3.2 (1.6, 4.8)	125	10.3 (7.9, 12.6)
Adjusted prevalence difference (95% CI; q-value)*	0.1 (-3.4, 3.6; 0.97)		-3.7 (-6.7, -0.7; 0.09)		3.7 (0.1, 7.3; 0.17)	
The United States needs a civil war to set things right.						
Do not agree	799	81.5 (77.8, 85.1)	879	83.6 (80.4, 86.7)	1159	76.2 (73.2, 79.2)
Somewhat agree	115	13.2 (10.1, 16.4)	118	13.1 (10.3, 16.0)	238	15.1 (12.7, 17.6)
Strongly or very strongly agree	29	3.8 (1.9, 5.6)	17	1.4 (0.6, 2.2)	88	6.9 (5.0, 8.8)
Adjusted prevalence difference (95% CI; q-value)*	0.3 (-2.4, 3.0; 0.92)		-2.2 (-4.2, -0.2; 0.13)		3.6 (0.8, 6.4; 0.08)	
Suppose a civil war occurred and took the form of a RIGHT-wing anti-government insurgency. Which side would you most likely support?						
Neither side	513	58.8 (54.6, 63.0)	597	61.5 (57.5, 65.6)	746	53.0 (49.5, 56.4)
The government	264	23.3 (19.8, 26.8)	297	25.2 (21.6, 28.8)	345	19.5 (16.8, 22.2)
The insurgency	157	15.8 (12.6, 18.9)	104	9.7 (7.2, 12.2)	377	24.5 (21.6, 27.4)
Adjusted prevalence difference (95% CI; q-value)†	13.2 (9.6, 16.9; < 0.001)		7.2 (4.1, 10.3; < 0.001)		22.1 (18.7, 25.6; < 0.001)	
Suppose a civil war occurred and took the form of a LEFT-wing anti-government insurgency. Which side would you most likely support?						
Neither side	496	58.8 (54.7, 63.0)	547	58.8 (54.8, 62.8)	708	51.7 (48.2, 55.1)
The government	415	36.6 (32.6, 40.7)	427	35.4 (31.5, 39.3)	682	39.6 (36.3, 42.9)
The insurgency	19	1.8 (0.8, 2.8)	26	2.7 (1.5, 4.0)	80	5.8 (4.3, 7.3)
Adjusted prevalence difference (95% CI; q-value)†	-1.3 (-3.1, 0.6; 0.42)		-0.1 (-2.1, 1.9; 0.97)		3.4 (1.2, 5.6; 0.02)	
If a civil war did occur, how likely would you be to do each of the following?						
Leave the United States						
Not likely	732	71.5 (67.4, 75.6)	792	72.6 (68.8, 76.4)	1279	81.2 (78.2, 84.1)
Somewhat likely	149	18.0 (14.5, 21.5)	170	19.9 (16.5, 23.4)	154	11.4 (9.2, 13.6)
Very or extremely likely	62	9.3 (6.6, 12.0)	50	5.5 (3.6, 7.4)	56	6.0 (3.8, 8.1)
Adjusted prevalence difference (95% CI; q-value)‡	-1.5 (-5.2, 2.3; 0.74)		-5.7 (-8.8, -2.6; 0.01)		-3.7 (-7.1, -0.4; 0.13)	
"Sit it out": stay in the United States, without participating in the conflict						
Not likely	132	12.4 (9.6, 15.2)	139	14.8 (11.6, 17.9)	275	17.6 (15.0, 20.1)
Somewhat likely	363	37.3 (33.1, 41.5)	370	33.3 (29.6, 37.1)	546	35.9 (32.6, 39.1)
Very or extremely likely	444	48.6 (44.3, 52.9)	498	49.6 (45.4, 53.7)	666	45.1 (41.7, 48.5)
Adjusted prevalence difference (95% CI; q-value)‡	-1.0 (-6.8, 4.9; 0.92)		0.8 (-4.9, 6.6; 0.92)		-4.2 (-9.6, 1.1; 0.35)	
Participate in a non-combat role (examples: providing food and supplies, working as a mechanic or medic)						
Not likely	319	34.9 (30.7, 39.0)	372	37.9 (33.8, 42.0)	471	33.4 (30.2, 36.7)
Somewhat likely	410	39.4 (35.3, 43.5)	409	37.7 (33.7, 41.6)	652	40.5 (37.2, 43.8)
Very or extremely likely	205	23.9 (19.9, 27.9)	224	21.8 (18.4, 25.3)	361	24.2 (21.2, 27.2)
Adjusted prevalence difference (95% CI; q-value)‡	5.1 (0.0, 10.1; 0.19)		4.0 (-0.8, 8.7; 0.32)		6.1 (1.7, 10.6; 0.06)	
Participate as a combatant (someone who is fighting)						
Not likely	742	80.8 (77.5, 84.1)	855	85.0 (82.1, 87.8)	1133	75.8 (72.8, 78.8)
Somewhat likely	148	13.5 (10.6, 16.4)	115	9.0 (6.8, 11.2)	249	14.8 (12.5, 17.1)
Very or extremely likely	47	4.0 (2.5, 5.5)	32	3.1 (1.7, 4.5)	98	7.4 (5.4, 9.4)
Adjusted prevalence difference (95% CI; q-value)‡	1.9 (-0.2, 4.0; 0.26)		1.2 (-0.8, 3.2; 0.5)		5.5 (2.9, 8.0; < 0.001)	
Kill a combatant from the opposing side						
Not likely	784	84.8 (81.8, 87.8)	892	87.6 (84.9, 90.3)	1207	81.0 (78.3, 83.8)
Somewhat likely	108	9.4 (6.9, 12.0)	85	7.4 (5.3, 9.4)	182	9.8 (8.0, 11.6)
Very or extremely likely	43	3.9 (2.4, 5.3)	28	2.5 (1.2, 3.8)	88	6.6 (4.6, 8.6)
Adjusted prevalence difference (95% CI; q-value)‡	1.3 (-0.8, 3.4; 0.5)		0.1 (-1.9, 2.2; 0.95)		4.3 (1.7, 6.8; 0.01)	
Kill a non-combatant from the opposing side						
Not likely	911	95.8 (94.1, 97.4)	978	94.8 (92.9, 96.6)	1405	90.9 (88.6, 93.3)
Somewhat likely	19	1.8 (0.7, 2.9)	17	1.5 (0.5, 2.5)	48	4.3 (2.6, 6.1)
Very or extremely likely	5	0.4 (0.0, 0.9)	6	0.4 (0.0, 0.8)	20	1.6 (0.6, 2.6)
Adjusted prevalence difference (95% CI; q-value)‡	-0.9 (-2.2, 0.3; 0.38)		-0.9 (-2.2, 0.3; 0.35)		0.4 (-1.2, 1.9; 0.88)	

Details of categorization for party affiliation are in the Additional Methods Text section of this supplement (p 12).

* Adjusted prevalence differences are for the strongly or very strongly agree comparison.

† Adjusted prevalence differences are for support of the insurgency (rather than the government or neither side).

‡ Adjusted prevalence differences are for the very or extremely likely option.

Adjusted models include age, race and ethnicity, gender, education, income, Census division, and rurality. Q-values represent the probability that the given difference would be a false discovery; they represent the expected proportion of “false positives” that would be seen among the collection of all differences whose q-values were at or below the given q-value.

Refusals were not included in the table but were included in calculating weighted percentages.

Table S4. Association between self-reported MAGA status and views on civil war in the United States

Query and Response	Republican				Non-Republican			
	MAGA Republican		Other Republican		MAGA Movement		Not MAGA Movement	
	Unweighted n	Weighted % (95% CI)	Unweighted n	Weighted % (95% CI)	Unweighted n	Weighted % (95% CI)	Unweighted n	Weighted % (95% CI)
How much do you agree or disagree with each of the following statements?[†]								
In the next few years, there will be civil war in the United States.								
Do not agree	653	51.2 (47.4, 55.0)	1513	65.6 (62.8, 68.4)	116	48.1 (38.7, 57.5)	2911	65.1 (63.2, 67.1)
Somewhat agree	468	37.4 (33.8, 41.0)	544	28.2 (25.6, 30.9)	50	24.1 (16.1, 32.1)	1229	28.3 (26.5, 30.1)
Strongly or very strongly agree	112	10.2 (7.6, 12.8)	87	5.3 (3.9, 6.7)	42	26.7 (17.7, 35.7)	178	5.5 (4.5, 6.6)
Adjusted prevalence difference (95% CI; q-value)*	6.4 (3.5, 9.2; < 0.001)		1.6 (-0.2, 3.5; 0.15)		20.0 (11.4, 28.7; < 0.001)		Referent	
The United States needs a civil war to set things right.								
Do not agree	894	72.3 (68.9, 75.6)	1888	84.9 (82.7, 87.2)	147	66.5 (57.2, 75.7)	4058	90.4 (89.0, 91.7)
Somewhat agree	256	19.1 (16.3, 21.8)	207	11.5 (9.4, 13.5)	31	12.5 (6.7, 18.4)	202	6.2 (5.1, 7.4)
Strongly or very strongly agree	86	7.8 (5.6, 10.1)	47	2.6 (1.6, 3.5)	31	20.0 (11.5, 28.6)	63	2.4 (1.7, 3.1)
Adjusted prevalence difference (95% CI; q-value)*	6.7 (4.2, 9.2; < 0.001)		1.5 (0.2, 2.7; 0.05)		16.6 (8.6, 24.6; < 0.001)		Referent	
Suppose a civil war occurred and took the form of a RIGHT-wing anti-government insurgency. Which side would you most likely support?								
Neither side	619	50.4 (46.7, 54.2)	1192	61.1 (58.4, 63.9)	124	59.2 (49.8, 68.6)	1847	51.3 (49.3, 53.3)
The government	220	16.4 (13.4, 19.5)	669	25.7 (23.3, 28.1)	55	26.9 (18.4, 35.4)	2362	44.8 (42.9, 46.8)
The insurgency	376	29.4 (26.0, 32.8)	256	11.4 (9.6, 13.2)	28	10.5 (4.2, 16.8)	90	2.5 (1.8, 3.1)
Adjusted prevalence difference (95% CI; q-value)[†]	27.9 (24.2, 31.6; < 0.001)		8.9 (7.0, 10.9; < 0.001)		8.1 (1.8, 14.3; 0.04)		Referent	
Suppose a civil war occurred and took the form of a LEFT-wing anti-government insurgency. Which side would you most likely support?								
Neither side	609	51.2 (47.4, 55.0)	1095	58.3 (55.5, 61.1)	117	58.0 (48.6, 67.4)	2235	58.9 (56.9, 60.8)
The government	545	39.9 (36.1, 43.6)	960	36.8 (34.1, 39.5)	70	26.1 (18.4, 33.7)	1722	31.1 (29.4, 32.8)
The insurgency	62	5.4 (3.8, 7.0)	61	3.0 (2.0, 3.9)	20	12.5 (4.7, 20.4)	334	8.4 (7.3, 9.5)
Adjusted prevalence difference (95% CI; q-value)[†]	-1.8 (-3.9, 0.3; 0.16)		-5.5 (-7.1, -3.9; < 0.001)		2.6 (-4.7, 10.0; 0.58)		Referent	
If a civil war did occur, how likely would you be to do each of the following?								
Leave the United States								
Not likely	1084	85.0 (82.0, 88.0)	1655	71.0 (68.2, 73.7)	139	55.4 (45.9, 64.9)	2436	52.3 (50.3, 54.3)
Somewhat likely	104	8.8 (6.7, 11.0)	362	20.1 (17.7, 22.5)	45	22.4 (15.0, 29.9)	1272	30.6 (28.7, 32.5)
Very or extremely likely	48	5.1 (2.9, 7.3)	118	7.7 (6.0, 9.3)	26	20.4 (11.7, 29.1)	604	15.8 (14.3, 17.3)
Adjusted prevalence difference (95% CI; q-value)[‡]	-7.1 (-9.8, -4.4; < 0.001)		-6.7 (-9.1, -4.4; < 0.001)		5.0 (-3.4, 13.3; 0.35)		Referent	
"Sit it out": stay in the United States, without participating in the conflict								
Not likely	249	18.9 (16.0, 21.8)	283	12.8 (10.8, 14.7)	33	13.0 (7.3, 18.8)	589	15.5 (13.9, 17.0)
Somewhat likely	473	38.0 (34.4, 41.7)	783	34.6 (31.9, 37.3)	82	38.3 (29.0, 47.5)	1560	34.4 (32.5, 36.3)
Very or extremely likely	511	41.8 (38.0, 45.6)	1062	51.2 (48.3, 54.0)	93	46.4 (37.1, 55.8)	2157	48.8 (46.8, 50.8)
Adjusted prevalence difference (95% CI; q-value)[‡]	-8.5 (-12.9, -4.1; < 0.001)		2.1 (-1.5, 5.8; 0.36)		-2.1 (-11.7, 7.4; 0.74)		Referent	
Participate in a non-combat role (examples: providing food and supplies, working as a mechanic or medic)								
Not likely	338	28.9 (25.4, 32.3)	799	38.8 (36.0, 41.7)	69	40.2 (30.8, 49.7)	1621	41.2 (39.2, 43.2)
Somewhat likely	551	41.2 (37.6, 44.9)	887	38.5 (35.7, 41.2)	78	32.6 (23.8, 41.5)	1724	36.7 (34.8, 38.6)
Very or extremely likely	342	28.5 (25.0, 32.1)	438	21.1 (18.7, 23.5)	60	23.8 (16.5, 31.1)	952	20.5 (18.9, 22.1)
Adjusted prevalence difference (95% CI; q-value)[‡]	8.9 (4.9, 12.9; < 0.001)		1.9 (-1.2, 4.9; 0.35)		3.2 (-4.4, 10.7; 0.51)		Referent	
Participate as a combatant (someone who is fighting)								
Not likely	898	72.6 (69.2, 75.9)	1774	84.9 (82.9, 86.9)	153	72.2 (63.1, 81.4)	3852	88.6 (87.3, 89.9)
Somewhat likely	228	16.8 (14.2, 19.5)	273	10.1 (8.5, 11.7)	34	12.8 (6.0, 19.5)	346	7.4 (6.3, 8.4)
Very or extremely likely	104	9.2 (6.8, 11.6)	72	3.1 (2.2, 4.1)	21	12.4 (5.2, 19.7)	108	2.6 (2.0, 3.3)
Adjusted prevalence difference (95% CI; q-value)[‡]	8.0 (5.5, 10.6; < 0.001)		1.9 (0.6, 3.1; 0.01)		9.1 (2.1, 16.2; 0.04)		Referent	
Kill a combatant from the opposing side								
Not likely	975	79.0 (75.9, 82.0)	1845	87.7 (85.9, 89.5)	164	79.3 (71.5, 87)	3921	90.8 (89.6, 91.9)
Somewhat likely	172	12.2 (10.0, 14.4)	200	7.5 (6.0, 9.0)	25	8.0 (4.2, 11.9)	270	5.2 (4.3, 6.0)
Very or extremely likely	81	7.3 (5.0, 9.6)	75	3.0 (2.1, 3.9)	18	10.2 (3.6, 16.8)	107	2.6 (1.9, 3.2)
Adjusted prevalence difference (95% CI; q-value)[‡]	5.7 (3.2, 8.2; < 0.001)		1.2 (0.0, 2.4; 0.11)		6.7 (0.3, 13.2; 0.10)		Referent	
Kill a non-combatant from the opposing side								
Not likely	1162	92.2 (89.9, 94.4)	2065	95.1 (93.7, 96.4)	186	85.3 (77.6, 92.9)	4180	94.7 (93.7, 95.7)
Somewhat likely	47	4.9 (2.9, 7.0)	35	1.7 (0.9, 2.4)	12	7.6 (1.2, 14.0)	76	2.6 (1.8, 3.3)
Very or extremely likely	18	1.2 (0.5, 1.8)	13	0.8 (0.2, 1.4)	10	4.7 (0.8, 8.6)	43	1.2 (0.8, 1.7)
Adjusted prevalence difference (95% CI; q-value)[‡]	0.6 (-0.2, 1.5; 0.25)		0.2 (-0.6, 1.0; 0.68)		2.9 (-1.0, 6.8; 0.23)		Referent	

Details of categorization for MAGA status are in the Additional Methods Text section of this supplement (p 12).

* Adjusted prevalence differences are for the strongly or very strongly agree comparison.

† Adjusted prevalence differences are for support of the insurgency (rather than the government or neither side).

‡ Adjusted prevalence differences are for the very or extremely likely option.

Adjusted models include age, race and ethnicity, gender, education, income, Census division, and rurality. Q-values represent the probability that the given difference would be a false discovery; they represent the expected proportion of “false positives” that would be seen among the collection of all differences whose q-values were at or below the given q-value.

Refusals were not included in the table but were included in calculating weighted percentages.

Table S5. Association between political ideology and views on civil war in the United States

Query and Response	Political Ideology							
	Extremely Liberal		Liberal		Slightly Liberal		Moderate/Middle of the Road	
	Unweighted n	Weighted % (95% CI)	Unweighted n	Weighted % (95% CI)	Unweighted n	Weighted % (95% CI)	Unweighted n	Weighted % (95% CI)
How much do you agree or disagree with each of the following statements?[†]								
In the next few years, there will be civil war in the United States.								
Do not agree	207	64.5 (57.4, 71.6)	769	67.0 (63.2, 70.8)	509	71.0 (66.7, 75.4)	1603	60.0 (57.4, 62.6)
Somewhat agree	91	28.4 (21.8, 35.0)	301	28.0 (24.4, 31.6)	183	23.6 (19.7, 27.5)	754	30.7 (28.3, 33.1)
Strongly or very strongly agree	17	5.1 (2.1, 8.2)	34	3.6 (2.0, 5.3)	22	4.1 (1.9, 6.3)	151	7.6 (6.0, 9.2)
Adjusted prevalence difference (95% CI; q-value)*	-2.3 (-6.0, 1.4; 0.5)		-2.9 (-5.2, -0.6; 0.08)		-2.6 (-5.3, 0.2; 0.28)		Referent	
The United States needs a civil war to set things right.								
Do not agree	290	87.6 (82.1, 93.1)	1056	91.9 (89.3, 94.4)	680	93.6 (91.0, 96.1)	2247	85.7 (83.7, 87.6)
Somewhat agree	17	7.0 (3.1, 10.9)	36	5.3 (3.2, 7.4)	23	3.0 (1.5, 4.5)	190	8.6 (7.0, 10.1)
Strongly or very strongly agree	9	3.8 (0.9, 6.6)	14	1.6 (0.4, 2.9)	12	2.3 (0.7, 4.0)	67	3.6 (2.6, 4.7)
Adjusted prevalence difference (95% CI; q-value)*	-0.5 (-3.9, 3.0; 0.88)		-1.2 (-2.8, 0.4; 0.42)		-0.8 (-2.8, 1.3; 0.68)		Referent	
Suppose a civil war occurred and took the form of a RIGHT-wing anti-government insurgency. Which side would you most likely support?								
Neither side	92	36.6 (29.2, 44.0)	330	37.1 (33.2, 40.9)	248	41.2 (36.2, 46.1)	1398	63.0 (60.6, 65.4)
The government	220	61.9 (54.5, 69.3)	744	57.9 (53.9, 61.8)	440	53.8 (48.9, 58.8)	991	30.3 (28.0, 32.5)
The insurgency	5	1.5 (0.0, 3.1)	22	2.7 (1.1, 4.2)	18	2.8 (1.0, 4.5)	101	4.3 (3.2, 5.3)
Adjusted prevalence difference (95% CI; q-value)[†]	-2.5 (-4.5, -0.5; 0.08)		-1.0 (-3.0, 1.1; 0.62)		-1.3 (-3.4, 0.8; 0.5)		Referent	
Suppose a civil war occurred and took the form of a LEFT-wing anti-government insurgency. Which side would you most likely support?								
Neither side	136	47.1 (39.8, 54.4)	513	52.3 (48.4, 56.1)	329	52.2 (47.3, 57.1)	1447	64.6 (62.2, 67.0)
The government	88	23.6 (17.6, 29.6)	453	32.0 (28.6, 35.4)	322	35.8 (31.3, 40.3)	948	29.2 (27.0, 31.4)
The insurgency	92	28.9 (22.5, 35.3)	128	13.4 (10.6, 16.3)	53	9.7 (6.4, 13.0)	94	3.6 (2.7, 4.6)
Adjusted prevalence difference (95% CI; q-value)[†]	21.7 (15.5, 27.9; < 0.001)		9.6 (6.5, 12.8; < 0.001)		5.5 (2.1, 8.9; 0.01)		Referent	
If a civil war did occur, how likely would you be to do each of the following?								
Leave the United States								
Not likely	134	38.9 (31.8, 45.9)	557	47.9 (44.0, 51.8)	362	45.6 (40.7, 50.4)	1668	61.4 (58.8, 64.0)
Somewhat likely	103	34.7 (27.7, 41.8)	372	32.3 (28.7, 35.9)	239	34.8 (30.1, 39.5)	596	25.8 (23.5, 28.2)
Very or extremely likely	78	26.0 (19.7, 32.3)	174	18.6 (15.4, 21.7)	112	18.0 (14.1, 21.9)	243	10.6 (9.0, 12.3)
Adjusted prevalence difference (95% CI; q-value)[‡]	12.5 (5.7, 19.2; 0.003)		7.0 (3.4, 10.6; 0.002)		6.4 (2.1, 10.7; 0.03)		Referent	
"Sit it out": stay in the United States, without participating in the conflict								
Not likely	56	19.1 (13.5, 24.8)	156	14.1 (11.3, 16.8)	63	10.4 (7.1, 13.7)	351	15.3 (13.3, 17.3)
Somewhat likely	109	31.8 (25.1, 38.5)	430	37.6 (33.9, 41.4)	266	34.3 (29.7, 38.8)	870	32.8 (30.3, 35.2)
Very or extremely likely	150	48.5 (41.2, 55.8)	518	47.0 (43.2, 50.9)	380	53.4 (48.5, 58.3)	1283	50.1 (47.5, 52.7)
Adjusted prevalence difference (95% CI; q-value)[‡]	-1.1 (-9.0, 6.8; 0.88)		-3.6 (-8.3, 1.2; 0.41)		3.5 (-2.2, 9.1; 0.5)		Referent	
Participate in a non-combat role (examples: providing food and supplies, working as a mechanic or medic)								
Not likely	100	34.1 (26.9, 41.4)	351	34.2 (30.5, 37.9)	240	35.9 (31.2, 40.7)	1014	43.7 (41.1, 46.4)
Somewhat likely	101	34.1 (27.2, 41.0)	479	39.9 (36.1, 43.7)	316	40.5 (35.8, 45.3)	981	35.9 (33.5, 38.4)
Very or extremely likely	114	31.2 (24.9, 37.5)	272	24.4 (21.1, 27.7)	155	21.8 (17.6, 26.0)	500	18.0 (16.0, 19.9)
Adjusted prevalence difference (95% CI; q-value)[‡]	12.4 (5.5, 19.3; 0.004)		5.8 (1.8, 9.7; 0.03)		3.7 (-1.0, 8.3; 0.38)		Referent	
Participate as a combatant (someone who is fighting)								
Not likely	268	84.9 (79.7, 90.1)	989	89.0 (86.4, 91.7)	645	87.9 (84.2, 91.5)	2197	87.5 (85.8, 89.2)
Somewhat likely	30	9.2 (4.9, 13.5)	85	6.8 (4.6, 8.9)	47	6.9 (4.3, 9.6)	237	8.1 (6.7, 9.5)
Very or extremely likely	16	5.1 (2.0, 8.2)	29	2.9 (1.4, 4.3)	19	3.3 (1.1, 5.5)	66	2.3 (1.6, 3.1)
Adjusted prevalence difference (95% CI; q-value)[‡]	2.2 (-1.2, 5.7; 0.47)		1.0 (-0.8, 2.7; 0.53)		1.2 (-1.1, 3.5; 0.54)		Referent	
Kill a combatant from the opposing side								
Not likely	277	86.9 (81.9, 91.9)	1019	92.3 (90.2, 94.4)	649	90.2 (87.1, 93.4)	2243	89.5 (87.9, 91.1)
Somewhat likely	21	7.5 (3.4, 11.7)	62	4.3 (2.8, 5.7)	46	5.2 (3.3, 7.1)	182	5.7 (4.6, 6.8)
Very or extremely likely	15	4.7 (1.7, 7.7)	23	2.2 (1.0, 3.4)	16	2.9 (0.7, 5.0)	75	2.8 (2.0, 3.6)
Adjusted prevalence difference (95% CI; q-value)[‡]	1.4 (-1.9, 4.7; 0.66)		-0.2 (-1.7, 1.3; 0.88)		0.2 (-2.1, 2.4; 0.92)		Referent	
Kill a non-combatant from the opposing side								
Not likely	300	92.8 (88.1, 97.6)	1083	96.0 (94.1, 97.8)	694	95.8 (93.5, 98.0)	2414	94.0 (92.7, 95.3)
Somewhat likely	5	3.5 (0.0, 7.6)	10	1.4 (0.3, 2.5)	9	1.2 (0.2, 2.1)	61	2.8 (1.9, 3.7)
Very or extremely likely	9	2.9 (0.3, 5.4)	11	1.4 (0.3, 2.4)	6	1.2 (0.0, 2.5)	24	1.0 (0.6, 1.5)
Adjusted prevalence difference (95% CI; q-value)[‡]	1.8 (-0.9, 4.4; 0.47)		0.6 (-0.7, 1.8; 0.62)		0.3 (-1.1, 1.7; 0.86)		Referent	

Table S5, continued.

Query and Response	Political Ideology					
	Slightly Conservative		Conservative		Extremely Conservative	
	Unweighted n	Weighted % (95% CI)	Unweighted n	Weighted %(95% CI)	Unweighted n	Weighted %(95% CI)
How much do you agree or disagree with each of the following statements?[†]						
In the next few years, there will be civil war in the United States.						
Do not agree	696	63.4 (59.1, 67.7)	1196	60.0 (56.9, 63.2)	225	51.4 (45.1, 57.8)
Somewhat agree	286	29.2 (25.2, 33.3)	535	30.0 (27.0, 33.0)	156	33.4 (27.5, 39.3)
Strongly or very strongly agree	38	6.5 (3.8, 9.3)	106	7.3 (5.4, 9.1)	53	13.0 (8.8, 17.2)
Adjusted prevalence difference (95% CI; q-value)*	0.4 (-2.7, 3.5; 0.88)		1.4 (-1.1, 3.8; 0.53)		6.5 (1.9, 11.0; 0.04)	
The United States needs a civil war to set things right.						
Do not agree	907	85.3 (81.7, 88.8)	1525	79.8 (77.1, 82.5)	309	68.3 (62.3, 74.3)
Somewhat agree	92	10.4 (7.4, 13.3)	258	14.3 (12.0, 16.6)	80	16.4 (11.8, 21.0)
Strongly or very strongly agree	23	3.8 (1.5, 6.1)	56	3.5 (2.1, 4.8)	46	13.0 (8.5, 17.6)
Adjusted prevalence difference (95% CI; q-value)*	1.1 (-1.4, 3.5; 0.65)		1.2 (-0.6, 2.9; 0.47)		10.6 (5.9, 15.3; < 0.001)	
Suppose a civil war occurred and took the form of a RIGHT-wing anti-government insurgency. Which side would you most likely support?						
Neither side	539	56.5 (52.3, 60.7)	968	55.8 (52.6, 58.9)	231	59.3 (53.2, 65.4)
The government	373	33.2 (29.1, 37.2)	470	21.0 (18.6, 23.4)	70	10.7 (7.4, 14.1)
The insurgency	100	8.8 (6.6, 10.9)	376	19.7 (17.1, 22.2)	132	26.9 (21.5, 32.3)
Adjusted prevalence difference (95% CI; q-value)[†]	4.6 (2.1, 7.1; 0.003)		15.7 (12.8, 18.7; < 0.001)		22.5 (16.9, 28.2; < 0.001)	
Suppose a civil war occurred and took the form of a LEFT-wing anti-government insurgency. Which side would you most likely support?						
Neither side	513	54.5 (50.3, 58.8)	937	56.3 (53.2, 59.4)	210	55.2 (49.0, 61.4)
The government	472	40.5 (36.3, 44.7)	819	37.1 (34.1, 40.0)	197	34.9 (29.1, 40.6)
The insurgency	26	3.4 (1.6, 5.2)	60	3.0 (2.0, 4.0)	25	6.2 (3.3, 9.1)
Adjusted prevalence difference (95% CI; q-value)[†]	-0.4 (-2.4, 1.5; 0.86)		-0.2 (-1.7, 1.3; 0.88)		2.7 (-0.5, 5.9; 0.33)	
If a civil war did occur, how likely would you be to do each of the following?						
Leave the United States						
Not likely	737	65.0 (60.7, 69.3)	1531	75.7 (72.7, 78.7)	381	82.3 (77.1, 87.5)
Somewhat likely	202	22.5 (18.8, 26.2)	220	14.1 (11.7, 16.5)	41	11.3 (7.0, 15.6)
Very or extremely likely	75	11.2 (8.0, 14.4)	92	8.1 (6.0, 10.3)	18	4.9 (2.0, 7.8)
Adjusted prevalence difference (95% CI; q-value)[‡]	0.6 (-3.0, 4.1; 0.88)		-0.4 (-3.1, 2.4; 0.88)		-4.0 (-7.4, -0.5; 0.13)	
"Sit it out": stay in the United States, without participating in the conflict						
Not likely	140	14.7 (11.4, 18.1)	297	14.9 (12.7, 17.2)	99	26.2 (20.2, 32.2)
Somewhat likely	370	35.6 (31.5, 39.7)	700	36.1 (33.1, 39.1)	156	34.1 (28.1, 40.0)
Very or extremely likely	501	48.1 (43.8, 52.3)	836	46.5 (43.3, 49.7)	183	38.1 (32.1, 44.1)
Adjusted prevalence difference (95% CI; q-value)[‡]	-2.1 (-7.2, 3.0; 0.66)		-3.6 (-7.8, 0.6; 0.33)		-12.8 (-19.4, -6.2; 0.002)	
Participate in a non-combat role (examples: providing food and supplies, working as a mechanic or medic)						
Not likely	401	41.4 (37.2, 45.7)	579	32.2 (29.3, 35.2)	139	35.5 (29.4, 41.7)
Somewhat likely	403	36.6 (32.5, 40.6)	803	39.9 (36.8, 43.0)	189	38.4 (32.3, 44.5)
Very or extremely likely	205	20.3 (16.8, 23.7)	442	24.9 (22.0, 27.7)	110	23.4 (18.0, 28.7)
Adjusted prevalence difference (95% CI; q-value)[‡]	2.4 (-1.6, 6.5; 0.5)		7.7 (4.0, 11.4; < 0.001)		5.5 (-0.4, 11.3; 0.28)	
Participate as a combatant (someone who is fighting)						
Not likely	836	82.5 (79.2, 85.8)	1448	78.3 (75.7, 80.9)	328	75.6 (70.1, 81.1)
Somewhat likely	138	11.1 (8.6, 13.6)	269	13.1 (11.0, 15.2)	80	14.8 (10.6, 18.9)
Very or extremely likely	37	4.8 (2.5, 7.0)	106	5.6 (4.1, 7.1)	29	6.9 (3.6, 10.3)
Adjusted prevalence difference (95% CI; q-value)[‡]	2.9 (0.5, 5.2; 0.09)		3.6 (1.9, 5.2; < 0.001)		4.7 (1.3, 8.1; 0.04)	
Kill a combatant from the opposing side						
Not likely	878	86.5 (83.5, 89.4)	1517	81.9 (79.5, 84.3)	355	80.8 (75.8, 85.9)
Somewhat likely	92	6.5 (4.8, 8.3)	204	9.6 (7.8, 11.3)	61	12.2 (8.3, 16.0)
Very or extremely likely	39	5.2 (2.8, 7.6)	96	5.2 (3.6, 6.7)	19	3.0 (1.4, 4.7)
Adjusted prevalence difference (95% CI; q-value)[‡]	2.8 (0.3, 5.3; 0.15)		3.0 (1.1, 4.9; 0.02)		0.3 (-1.7, 2.2; 0.88)	
Kill a non-combatant from the opposing side						
Not likely	974	92.0 (89.0, 95.1)	1750	92.1 (90.3, 93.9)	412	89.1 (84.4, 93.8)
Somewhat likely	26	5.0 (2.2, 7.8)	46	3.0 (1.7, 4.2)	16	4.7 (1.8, 7.5)
Very or extremely likely	8	0.8 (0.0, 1.7)	22	1.4 (0.6, 2.2)	3	0.6 (0.0, 1.3)
Adjusted prevalence difference (95% CI; q-value)[‡]	0.1 (-0.9, 1.1; 0.89)		0.9 (-0.2, 1.9; 0.33)		-0.1 (-1.1, 0.8; 0.88)	

Details of categorization for political ideology are in the Additional Methods Text section of this supplement (p 12).

* Adjusted prevalence differences are for the strongly or very strongly agree comparison.

† Adjusted prevalence differences are for support of the insurgency (rather than the government or neither side).

‡ Adjusted prevalence differences are for the very or extremely likely option.

Adjusted models include age, race and ethnicity, gender, education, income, Census division, and rurality. Q-values represent the probability that the given difference would be a false discovery; they represent the expected proportion of “false positives” that would be seen among the collection of all differences whose q-values were at or below the given q-value.

Refusals were not included in the table but were included in calculating weighted percentages.

Table S6. Association between beliefs on democracy and authoritarianism and views on civil war in the United States

Query and Response	Having a Strong Leader for America Is More Important than Having a Democracy					
	Do Not Agree		Somewhat Agree		Strongly or very Strongly Agree	
	Unweighted n	Weighted % (95% CI)	Unweighted n	Weighted % (95% CI)	Unweighted n	Weighted % (95% CI)
How much do you agree or disagree with each of the following statements?[†]						
In the next few years, there will be civil war in the United States.						
Do not agree	3984	71.4 (69.8, 73.0)	735	53.3 (49.6, 57.1)	531	39.9 (36.1, 43.6)
Somewhat agree	1412	24.4 (22.9, 25.9)	469	37.7 (34.1, 41.3)	441	39.6 (35.7, 43.5)
Strongly or very strongly agree	172	3.4 (2.7, 4.1)	81	7.2 (5.2, 9.2)	172	18.7 (15.3, 22.1)
Adjusted prevalence difference (95% CI; q-value)*	Referent		2.5 (0.3, 4.7; 0.08)		14.2 (10.8, 17.5; < 0.001)	
The United States needs a civil war to set things right.						
Do not agree	5229	93.0 (92.1, 94.0)	1037	78.5 (75.4, 81.6)	800	66.5 (62.7, 70.4)
Somewhat agree	273	4.8 (4.0, 5.6)	202	15.1 (12.4, 17.7)	229	19.8 (16.6, 23.1)
Strongly or very strongly agree	71	1.5 (1.0, 1.9)	49	4.5 (3.0, 6.0)	112	11.4 (8.7, 14.2)
Adjusted prevalence difference (95% CI; q-value)*	Referent		2.4 (0.7, 4.0; 0.02)		9.5 (6.8, 12.2; < 0.001)	
Suppose a civil war occurred and took the form of a RIGHT-wing anti-government insurgency. Which side would you most likely support?						
Neither side	2357	46.9 (45.2, 48.7)	788	67.5 (64.2, 70.8)	691	64.7 (61.0, 68.5)
The government	2756	44.8 (43.1, 46.5)	318	20.5 (17.6, 23.4)	261	19.2 (16.2, 22.2)
The insurgency	402	6.3 (5.5, 7.1)	169	9.5 (7.7, 11.3)	183	14.1 (11.3, 16.8)
Adjusted prevalence difference (95% CI; q-value)[†]	Referent		3.9 (1.7, 6.1; 0.002)		8.0 (5.1, 10.9; < 0.001)	
Suppose a civil war occurred and took the form of a LEFT-wing anti-government insurgency. Which side would you most likely support?						
Neither side	2582	51.8 (50.0, 53.6)	802	68.9 (65.6, 72.2)	734	68.9 (65.4, 72.4)
The government	2577	38.4 (36.8, 40.1)	409	24.1 (21.0, 27.1)	331	22.9 (19.7, 26.0)
The insurgency	354	7.8 (6.8, 8.9)	58	3.9 (2.7, 5.2)	70	6.3 (4.4, 8.2)
Adjusted prevalence difference (95% CI; q-value)[†]	Referent		-3.8 (-5.6, -1.9; < 0.001)		-0.8 (-3.1, 1.4; 0.63)	
If a civil war did occur, how likely would you be to do each of the following?						
Leave the United States						
Not likely	3644	59.5 (57.7, 61.2)	901	62.0 (58.3, 65.7)	855	66.3 (62.4, 70.2)
Somewhat likely	1341	26.6 (25.0, 28.1)	278	26.0 (22.6, 29.5)	173	18.1 (14.8, 21.4)
Very or extremely likely	566	12.6 (11.4, 13.9)	109	10.5 (8.1, 12.8)	123	14.8 (11.7, 17.9)
Adjusted prevalence difference (95% CI; q-value)[‡]	Referent		-2.7 (-5.4, 0.1; 0.15)		2.9 (-0.3, 6.2; 0.16)	
"Sit it out": stay in the United States, without participating in the conflict						
Not likely	759	13.7 (12.5, 14.9)	188	18.6 (15.4, 21.8)	226	18.4 (15.3, 21.4)
Somewhat likely	2030	35.1 (33.5, 36.8)	519	37.2 (33.7, 40.7)	377	31.3 (27.6, 35.0)
Very or extremely likely	2749	49.7 (47.9, 51.4)	582	43.1 (39.4, 46.7)	543	49.2 (45.3, 53.1)
Adjusted prevalence difference (95% CI; q-value)[‡]	Referent		-6.7 (-10.9, -2.6; 0.01)		-0.7 (-5.1, 3.8; 0.86)	
Participate in a non-combat role (examples: providing food and supplies, working as a mechanic or medic)						
Not likely	1988	38.7 (36.9, 40.4)	455	38.2 (34.5, 41.8)	411	40.3 (36.4, 44.2)
Somewhat likely	2290	38.3 (36.6, 40.0)	564	38.9 (35.3, 42.5)	433	33.7 (30.1, 37.4)
Very or extremely likely	1248	21.2 (19.8, 22.7)	263	21.2 (18.2, 24.3)	301	24.5 (21.2, 27.9)
Adjusted prevalence difference (95% CI; q-value)[‡]	Referent		-0.2 (-3.6, 3.2; 0.91)		3.4 (-0.3, 7.1; 0.15)	
Participate as a combatant (someone who is fighting)						
Not likely	4820	88.2 (87.1, 89.3)	1061	80.8 (77.8, 83.8)	881	78.4 (75.2, 81.6)
Somewhat likely	542	7.7 (6.8, 8.6)	171	12.5 (10.0, 14.9)	177	12.8 (10.2, 15.4)
Very or extremely likely	170	2.5 (2.0, 3.0)	50	5.1 (3.2, 6.9)	85	7.3 (5.3, 9.4)
Adjusted prevalence difference (95% CI; q-value)[‡]	Referent		1.7 (-0.1, 3.4; 0.15)		4.4 (2.3, 6.6; < 0.001)	
Kill a combatant from the opposing side						
Not likely	4945	90.6 (89.6, 91.5)	1109	85.3 (82.7, 87.9)	936	81.2 (78.0, 84.3)
Somewhat likely	434	5.7 (5.0, 6.4)	114	8.0 (6.2, 9.9)	126	9.5 (7.2, 11.8)
Very or extremely likely	145	2.0 (1.5, 2.4)	57	5.2 (3.4, 7.0)	83	7.7 (5.5, 9.9)
Adjusted prevalence difference (95% CI; q-value)[‡]	Referent		3.1 (1.2, 4.9; 0.01)		5.7 (3.4, 7.9; < 0.001)	
Kill a non-combatant from the opposing side						
Not likely	5415	96.4 (95.7, 97.1)	1209	90.7 (88.4, 93.0)	1057	88.2 (85.3, 91.1)
Somewhat likely	71	1.1 (0.8, 1.5)	54	6.0 (4.0, 8.0)	51	6.4 (4.0, 8.8)
Very or extremely likely	35	0.5 (0.3, 0.7)	14	1.4 (0.5, 2.3)	36	3.7 (2.2, 5.3)
Adjusted prevalence difference (95% CI; q-value)[‡]	Referent		0.6 (-0.4, 1.5; 0.42)		3.0 (1.5, 4.5; < 0.001)	

Table S6, continued.

Query and Response	We Should Suspend Congress for a Few Years So a Strong Leader Can Clean Up the Mess Made by Politicians in Washington					
	Do Not Agree		Somewhat Agree		Strongly or very Strongly Agree	
	Unweighted n	Weighted % (95% CI)	Unweighted n	Weighted % (95% CI)	Unweighted n	Weighted % (95% CI)
How much do you agree or disagree with each of the following statements?[†]						
In the next few years, there will be civil war in the United States.						
Do not agree	4238	73.2 (71.7, 74.8)	627	48.0 (44.3, 51.8)	389	34.5 (30.7, 38.3)
Somewhat agree	1370	23.2 (21.7, 24.6)	522	42.5 (38.8, 46.2)	438	41.0 (37.0, 45.1)
Strongly or very strongly agree	141	2.7 (2.1, 3.2)	76	6.5 (4.6, 8.4)	207	23.6 (19.8, 27.4)
Adjusted prevalence difference (95% CI; q-value)*	Referent		2.9 (0.8, 5.1; 0.03)		19.8 (16.1, 23.4; < 0.001)	
The United States needs a civil war to set things right.						
Do not agree	5432	93.7 (92.8, 94.5)	983	76.6 (73.3, 80.0)	666	64.0 (59.9, 68.0)
Somewhat agree	274	4.6 (3.9, 5.4)	199	16.5 (13.6, 19.4)	232	19.9 (16.6, 23.1)
Strongly or very strongly agree	52	0.9 (0.6, 1.2)	43	3.7 (2.3, 5.1)	136	15.4 (12.2, 18.6)
Adjusted prevalence difference (95% CI; q-value)*	Referent		2.5 (1.0, 4.0; 0.01)		14.0 (11.0, 17.1; < 0.001)	
Suppose a civil war occurred and took the form of a RIGHT-wing anti-government insurgency. Which side would you most likely support?						
Neither side	2399	46.3 (44.6, 48.1)	798	70.0 (66.7, 73.3)	656	66.9 (63.0, 70.7)
The government	2856	44.9 (43.2, 46.6)	285	19.3 (16.4, 22.1)	193	17.4 (14.2, 20.5)
The insurgency	443	6.8 (6.0, 7.7)	136	8.0 (6.2, 9.9)	176	14.1 (11.4, 16.8)
Adjusted prevalence difference (95% CI; q-value)[†]	Referent		1.9 (-0.3, 4.1; 0.23)		7.9 (4.9, 10.9; < 0.001)	
Suppose a civil war occurred and took the form of a LEFT-wing anti-government insurgency. Which side would you most likely support?						
Neither side	2611	51.1 (49.4, 52.9)	828	72.2 (69.0, 75.4)	694	70.0 (66.2, 73.7)
The government	2736	39.5 (37.8, 41.1)	329	20.1 (17.3, 22.8)	254	21.0 (17.7, 24.3)
The insurgency	343	7.3 (6.3, 8.3)	64	5.1 (3.5, 6.6)	75	6.9 (4.8, 9.0)
Adjusted prevalence difference (95% CI; q-value)[†]	Referent		-1.8 (-3.8, 0.2; 0.23)		0.3 (-2.1, 2.6; 0.9)	
If a civil war did occur, how likely would you be to do each of the following?						
Leave the United States						
Not likely	3818	60.0 (58.3, 61.8)	855	61.0 (57.3, 64.8)	739	65.3 (61.2, 69.4)
Somewhat likely	1351	26.1 (24.5, 27.7)	271	26.1 (22.6, 29.5)	177	20.1 (16.5, 23.6)
Very or extremely likely	573	12.6 (11.3, 13.9)	107	11.2 (8.8, 13.6)	119	13.9 (10.8, 16.9)
Adjusted prevalence difference (95% CI; q-value)[‡]	Referent		-1.9 (-4.8, 1.0; 0.36)		1.7 (-1.5, 5.0; 0.4)	
"Sit it out": stay in the United States, without participating in the conflict						
Not likely	774	14.0 (12.7, 15.2)	200	17.5 (14.5, 20.6)	201	18.6 (15.4, 21.9)
Somewhat likely	2119	35.2 (33.6, 36.9)	488	38.9 (35.3, 42.6)	330	29.3 (25.6, 32.9)
Very or extremely likely	2830	49.3 (47.5, 51.0)	547	42.3 (38.6, 46.0)	503	51.3 (47.1, 55.4)
Adjusted prevalence difference (95% CI; q-value)[‡]	Referent		-7.0 (-11.2, -2.7; 0.01)		1.6 (-3.1, 6.2; 0.61)	
Participate in a non-combat role (examples: providing food and supplies, working as a mechanic or medic)						
Not likely	2046	38.9 (37.2, 40.7)	463	40.2 (36.5, 43.9)	352	37.0 (33.0, 41.0)
Somewhat likely	2403	38.8 (37.1, 40.4)	517	37.8 (34.2, 41.4)	374	32.9 (29.1, 36.8)
Very or extremely likely	1266	20.7 (19.3, 22.0)	249	20.1 (17.1, 23.2)	300	28.3 (24.6, 32.0)
Adjusted prevalence difference (95% CI; q-value)[‡]	Referent		0.1 (-3.4, 3.6; 0.95)		8.4 (4.4, 12.4; < 0.001)	
Participate as a combatant (someone who is fighting)						
Not likely	4946	87.5 (86.4, 88.6)	1030	82.8 (79.9, 85.7)	798	77.8 (74.3, 81.2)
Somewhat likely	601	8.2 (7.4, 9.1)	153	12.4 (9.8, 14.9)	140	11.1 (8.6, 13.6)
Very or extremely likely	169	2.7 (2.1, 3.2)	46	3.1 (1.7, 4.5)	91	9.6 (7.1, 12.1)
Adjusted prevalence difference (95% CI; q-value)[‡]	Referent		-0.3 (-1.8, 1.1; 0.73)		6.6 (4.2, 9.1; < 0.001)	
Kill a combatant from the opposing side						
Not likely	5097	90.2 (89.3, 91.2)	1069	86.3 (83.6, 88.9)	841	80.8 (77.6, 84.1)
Somewhat likely	452	5.7 (5.0, 6.4)	118	8.7 (6.5, 10.8)	106	9.1 (6.9, 11.3)
Very or extremely likely	162	2.4 (1.9, 3.0)	43	3.4 (2.0, 4.9)	78	8.1 (5.7, 10.5)
Adjusted prevalence difference (95% CI; q-value)[‡]	Referent		0.9 (-0.7, 2.4; 0.39)		5.7 (3.2, 8.2; < 0.001)	
Kill a non-combatant from the opposing side						
Not likely	5599	96.2 (95.5, 96.9)	1155	91.8 (89.6, 94.0)	944	87.1 (84.0, 90.2)
Somewhat likely	76	1.4 (1.0, 1.9)	54	4.7 (2.9, 6.5)	46	7.3 (4.7, 9.8)
Very or extremely likely	32	0.5 (0.3, 0.8)	17	1.6 (0.6, 2.5)	36	3.7 (2.2, 5.2)
Adjusted prevalence difference (95% CI; q-value)[‡]	Referent		0.7 (-0.4, 1.7; 0.38)		2.9 (1.5, 4.4; < 0.001)	

* Adjusted prevalence differences are for the strongly or very strongly agree comparison.

† Adjusted prevalence differences are for support of the insurgency (rather than the government or neither side).

‡ Adjusted prevalence differences are for the very or extremely likely option.

Adjusted models include age, race and ethnicity, gender, education, income, Census division, and rurality. Q-values represent the probability that the given difference would be a false discovery; they represent the expected proportion of "false positives" that would be seen among the collection of all differences whose q-values were at or below the given q-value.

Refusals were not included in the table but were included in calculating weighted percentages.

Table S7. Association between beliefs about race and ethnicity and views on civil war in the United States

Query and Response	Beliefs about Race and Ethnicity							
	Non-Agreement		Weak Agreement		Moderate Agreement		Strong Agreement	
	Unweighted n	Weighted % (95% CI)	Unweighted n	Weighted % (95% CI)	Unweighted n	Weighted % (95% CI)	Unweighted n	Weighted % (95% CI)
How much do you agree or disagree with each of the following statements?†								
In the next few years, there will be civil war in the United States.								
Do not agree	1030	69.9 (66.8, 73.1)	1343	67.5 (64.6, 70.4)	1762	60.9 (58.3, 63.5)	1116	51.5 (48.5, 54.5)
Somewhat agree	376	25.1 (22.3, 28.0)	493	27.5 (24.7, 30.2)	739	29.8 (27.3, 32.2)	724	36.5 (33.6, 39.4)
Strongly or very strongly agree	49	4.5 (2.8, 6.2)	48	3.3 (2.2, 4.5)	154	8.1 (6.5, 9.8)	175	10.4 (8.3, 12.5)
Adjusted prevalence difference (95% CI; q-value)*	Referent		-1.4 (-3.5, 0.6; 0.28)		3.2 (0.9, 5.6; 0.02)		7.5 (4.6, 10.3; <0.001)	
The United States needs a civil war to set things right.								
Do not agree	1415	95.6 (94.1, 97.2)	1806	92.8 (91.0, 94.6)	2314	82.0 (79.8, 84.2)	1539	73.7 (70.9, 76.4)
Somewhat agree	36	3.6 (2.2, 5.1)	68	4.5 (3.1, 5.9)	243	11.0 (9.2, 12.8)	361	18.0 (15.7, 20.2)
Strongly or very strongly agree	7	0.5 (0.0, 0.9)	12	0.9 (0.3, 1.5)	99	5.8 (4.4, 7.2)	112	6.5 (4.8, 8.2)
Adjusted prevalence difference (95% CI; q-value)*	Referent		0.4 (-0.4, 1.2; 0.44)		5.5 (3.9, 7.1; <0.001)		7.6 (5.4, 9.7; <0.001)	
Suppose a civil war occurred and took the form of a RIGHT-wing anti-government insurgency. Which side would you most likely support?								
Neither side	441	36.7 (33.4, 40.0)	802	51.7 (48.7, 54.7)	1520	63.1 (60.7, 65.6)	1081	57.6 (54.8, 60.5)
The government	984	60.5 (57.1, 63.8)	1029	43.7 (40.8, 46.7)	895	26.7 (24.5, 28.8)	427	17.1 (15.0, 19.3)
The insurgency	23	1.7 (0.8, 2.6)	40	2.8 (1.7, 3.9)	218	8.2 (6.7, 9.7)	476	21.7 (19.4, 24.0)
Adjusted prevalence difference (95% CI; q-value)†	Referent		1.2 (-0.3, 2.8; 0.24)		6.4 (4.4, 8.3; <0.001)		20.5 (17.7, 23.3; <0.001)	
Suppose a civil war occurred and took the form of a LEFT-wing anti-government insurgency. Which side would you most likely support?								
Neither side	669	52.0 (48.7, 55.4)	937	57.8 (54.8, 60.7)	1472	61.9 (59.5, 64.4)	1048	57.2 (54.3, 60.1)
The government	574	30.4 (27.6, 33.3)	821	34.7 (31.9, 37.5)	1077	32.7 (30.4, 35.1)	851	34.2 (31.5, 36.9)
The insurgency	203	16.3 (13.7, 19.0)	109	5.6 (4.3, 7.0)	84	3.2 (2.3, 4.0)	86	5.2 (3.7, 6.6)
Adjusted prevalence difference (95% CI; q-value)†	Referent		-9.4 (-12.4, -6.5; <0.001)		-12.3 (-15.1, -9.4; <0.001)		-9.9 (-13.0, -6.8; <0.001)	
If a civil war did occur, how likely would you be to do each of the following?								
Leave the United States								
Not likely	649	40.2 (37.0, 43.4)	1119	55.6 (52.5, 58.6)	1939	66.1 (63.6, 68.7)	1701	80.3 (77.7, 82.8)
Somewhat likely	516	36.3 (33.1, 39.5)	542	30.1 (27.3, 32.9)	503	22.1 (19.8, 24.4)	236	12.7 (10.7, 14.6)
Very or extremely likely	285	22.7 (19.8, 25.7)	219	13.1 (11.0, 15.1)	217	10.4 (8.7, 12.1)	79	5.5 (3.7, 7.2)
Adjusted prevalence difference (95% CI; q-value)‡	Referent		-8.5 (-12.1, -4.8; <0.001)		-10.0 (-13.5, -6.5; <0.001)		-12.9 (-16.6, -9.2; <0.001)	
"Sit it out": stay in the United States, without participating in the conflict								
Not likely	209	14.1 (11.7, 16.4)	211	11.3 (9.3, 13.2)	382	17.7 (15.5, 19.9)	370	17.8 (15.5, 20.0)
Somewhat likely	523	34.2 (31.1, 37.4)	701	36.2 (33.3, 39.2)	965	34.5 (32.1, 37.0)	749	35.2 (32.4, 38.0)
Very or extremely likely	719	51.0 (47.7, 54.4)	966	51.2 (48.1, 54.2)	1297	46.2 (43.7, 48.8)	893	45.3 (42.4, 48.3)
Adjusted prevalence difference (95% CI; q-value)‡	Referent		-0.5 (-5.1, 4.1; 0.85)		-5.6 (-10.0, -1.2; 0.03)		-7.2 (-12.1, -2.4; 0.01)	
Participate in a non-combat role (examples: providing food and supplies, working as a mechanic or medic)								
Not likely	477	37.1 (33.7, 40.4)	716	41.3 (38.3, 44.4)	981	39.8 (37.2, 42.4)	677	35.1 (32.3, 38.0)
Somewhat likely	562	35.7 (32.5, 38.8)	780	38.1 (35.2, 41.0)	1120	38.7 (36.2, 41.2)	837	38.2 (35.3, 41.0)
Very or extremely likely	409	26.3 (23.4, 29.2)	380	19.1 (16.8, 21.5)	538	19.7 (17.6, 21.8)	491	24.3 (21.7, 26.9)
Adjusted prevalence difference (95% CI; q-value)‡	Referent		-6.7 (-10.5, -3.0; 0.002)		-6.2 (-10.0, -2.5; 0.004)		-1.1 (-5.3, 3.0; 0.7)	
Participate as a combatant (someone who is fighting)								
Not likely	1322	91.9 (90.2, 93.5)	1691	90.3 (88.6, 92.1)	2219	82.6 (80.5, 84.6)	1532	75.9 (73.3, 78.5)
Somewhat likely	95	5.6 (4.2, 7.0)	153	6.8 (5.3, 8.3)	312	10.3 (8.7, 11.9)	336	15.4 (13.2, 17.5)
Very or extremely likely	32	1.6 (0.8, 2.3)	33	1.5 (0.9, 2.2)	112	5.4 (4.1, 6.7)	130	6.3 (4.7, 7.9)
Adjusted prevalence difference (95% CI; q-value)‡	Referent		0.1 (-1.0, 1.3; 0.85)		3.8 (2.2, 5.5; <0.001)		6.1 (4.1, 8.1; <0.001)	
Kill a combatant from the opposing side								
Not likely	1336	93.5 (92.1, 94.9)	1730	91.8 (90.1, 93.5)	2321	86.9 (85.1, 88.6)	1611	79.5 (77.1, 82.0)
Somewhat likely	88	4.6 (3.4, 5.8)	108	4.9 (3.6, 6.3)	219	7.0 (5.8, 8.2)	259	10.8 (9.0, 12.5)
Very or extremely likely	24	1.0 (0.5, 1.5)	37	1.7 (1.0, 2.5)	99	4.4 (3.2, 5.6)	126	6.8 (5.1, 8.6)
Adjusted prevalence difference (95% CI; q-value)‡	Referent		1.0 (-0.1, 2.0; 0.15)		3.7 (2.3, 5.2; <0.001)		7.2 (4.9, 9.4; <0.001)	
Kill a non-combatant from the opposing side								
Not likely	1434	97.8 (96.7, 98.9)	1837	96.4 (95.2, 97.6)	2511	91.5 (89.9, 93.1)	1911	91.3 (89.2, 93.4)
Somewhat likely	10	0.8 (0.1, 1.5)	22	1.4 (0.6, 2.3)	85	4.7 (3.4, 5.9)	58	3.8 (2.2, 5.4)
Very or extremely likely	5	0.5 (0.0, 1.1)	13	0.7 (0.2, 1.1)	44	1.9 (1.2, 2.6)	23	1.5 (0.6, 2.4)
Adjusted prevalence difference (95% CI; q-value)‡	Referent		0.1 (-0.7, 0.9; 0.85)		1.5 (0.5, 2.5; 0.01)		1.7 (0.5, 3.0; 0.02)	

Details of categorization for beliefs about race and ethnicity are in the Additional Methods Text section of this supplement (p 13).

* Adjusted prevalence differences are for the strongly or very strongly agree comparison.

† Adjusted prevalence differences are for support of the insurgency (rather than the government or neither side).

‡ Adjusted prevalence differences are for the very or extremely likely option.

Adjusted models include age, race and ethnicity, gender, education, income, Census division, and rurality. Q-values represent the probability that the given difference would be a false discovery; they represent the expected proportion of “false positives” that would be seen among the collection of all differences whose q-values were at or below the given q-value.

Refusals were not included in the table but were included in calculating weighted percentages.

Table S8. Association between beliefs about violence to effect social change and views on civil war in the United States

Query and Response	Beliefs about Violence to Effect Social Change							
	Non-Agreement		Weak Agreement		Moderate Agreement		Strong Agreement	
	Unweighted n	Weighted % (95% CI)	Unweighted n	Weighted % (95% CI)	Unweighted n	Weighted % (95% CI)	Unweighted n	Weighted % (95% CI)
How much do you agree or disagree with each of the following statements?[†]								
In the next few years, there will be civil war in the United States.								
Do not agree	3144	74.3 (72.6, 76.1)	1355	61.5 (58.6, 64.4)	593	42.8 (39.0, 46.6)	174	30.3 (25.2, 35.4)
Somewhat agree	912	22.2 (20.5, 23.9)	625	31.1 (28.4, 33.9)	557	44.1 (40.3, 48.0)	241	40.3 (34.7, 45.8)
Strongly or very strongly agree	82	2.4 (1.7, 3.0)	79	5.7 (4.1, 7.3)	115	11.8 (9.0, 14.5)	149	27.5 (22.3, 32.7)
Adjusted prevalence difference (95% CI; q-value)*	Referent		2.9 (1.1, 4.6; 0.004)		8.1 (5.4, 10.9; < 0.001)		23.7 (18.5, 29.0; < 0.001)	
The United States needs a civil war to set things right.								
Do not agree	4041	95.9 (94.9, 96.8)	1883	89.4 (87.4, 91.3)	892	65.9 (62.0, 69.7)	278	49.8 (44.2, 55.5)
Somewhat agree	92	3.0(2.2, 3.8)	153	7.7 (6.1, 9.3)	298	23.7 (20.3, 27.1)	164	23.8 (19.3, 28.2)
Strongly or very strongly agree	15	0.4 (0.1, 0.6)	28	1.5 (0.8, 2.2)	67	8.1 (5.6, 10.5)	120	23.8 (18.8, 28.8)
Adjusted prevalence difference (95% CI; q-value)*	Referent		0.7 (-0.1, 1.4; 0.14)		7.2 (4.9, 9.5; < 0.001)		22.8 (17.8, 27.8; < 0.001)	
Suppose a civil war occurred and took the form of a RIGHT-wing anti-government insurgency. Which side would you most likely support?								
Neither side	1757	48.6 (46.6, 50.6)	1109	59.5 (56.6, 62.3)	718	61.8 (58.0, 65.5)	272	51.2 (45.6, 56.9)
The government	2204	46.2 (44.2, 48.2)	758	31.2 (28.6, 33.8)	285	20.2 (17.1, 23.4)	92	16.0 (11.6, 20.4)
The insurgency	141	3.1 (2.5, 3.8)	169	6.9 (5.5, 8.3)	253	16.1 (13.4, 18.8)	195	29.6 (24.6, 34.6)
Adjusted prevalence difference (95% CI; q-value)[†]	Referent		4.1 (2.5, 5.7; < 0.001)		13.6 (10.8, 16.4; < 0.001)		27.9 (22.8, 33.0; < 0.001)	
Suppose a civil war occurred and took the form of a LEFT-wing anti-government insurgency. Which side would you most likely support?								
Neither side	1989	55.0 (53.0, 57.0)	1126	60.3 (57.5, 63.2)	714	63.2 (59.6, 66.9)	311	57.0 (51.4, 62.6)
The government	1902	37.7 (35.8, 39.5)	783	29.3 (26.8, 31.8)	448	26.8 (23.4, 30.1)	193	28.9 (23.9, 33.9)
The insurgency	204	5.1 (4.3, 6.0)	129	8.0 (6.2, 9.7)	91	7.8 (5.8, 9.9)	57	11.6 (7.6, 15.6)
Adjusted prevalence difference (95% CI; q-value)[†]	Referent		2.6 (0.6, 4.5; 0.02)		3.0 (0.7, 5.2; 0.02)		7.0 (2.9, 11.0; 0.003)	
If a civil war did occur, how likely would you be to do each of the following?								
Leave the United States								
Not likely	2631	59.1 (57.0, 61.1)	1432	60.9 (57.9, 63.9)	933	64.3 (60.4, 68.2)	427	66.9 (61.2, 72.5)
Somewhat likely	1048	26.5 (24.7, 28.3)	441	25.5 (22.8, 28.2)	231	23.1 (19.6, 26.6)	80	17.9 (13.2, 22.7)
Very or extremely likely	448	13.0 (11.5, 14.4)	190	12.5 (10.4, 14.6)	106	11.3 (8.6, 14.0)	58	12.9 (8.8, 17.0)
Adjusted prevalence difference (95% CI; q-value)[‡]	Referent		-0.2 (-2.8, 2.4; 0.87)		-1.0 (-4.1, 2.0; 0.65)		1.0 (-3.5, 5.4; 0.75)	
"Sit it out": stay in the United States, without participating in the conflict								
Not likely	462	11.9 (10.5, 13.3)	300	15.4 (13.2, 17.6)	254	21.4 (18.1, 24.7)	161	25.3 (20.4, 30.1)
Somewhat likely	1435	33.7 (31.8, 35.6)	778	35.6 (32.8, 38.5)	534	38.7 (35.0, 42.5)	194	31.5 (26.4, 36.6)
Very or extremely likely	2221	52.8 (50.8, 54.8)	980	47.9 (44.9, 50.8)	478	38.5 (34.7, 42.3)	209	40.9 (35.3, 46.5)
Adjusted prevalence difference (95% CI; q-value)[‡]	Referent		-5.6 (-9.3, -2.0; 0.01)		-15.2 (-19.6, -10.8; < 0.001)		-12.7 (-18.7, -6.6; < 0.001)	
Participate in a non-combat role (examples: providing food and supplies, working as a mechanic or medic)								
Not likely	1606	41.5 (39.5, 43.5)	692	36.8 (33.8, 39.7)	388	34.4 (30.6, 38.1)	169	35.0 (29.5, 40.5)
Somewhat likely	1666	37.5 (35.6, 39.5)	881	38.6 (35.7, 41.4)	564	40.5 (36.7, 44.3)	197	30.1 (25.0, 35.2)
Very or extremely likely	833	19.0 (17.4, 20.5)	478	23.2 (20.7, 25.7)	311	23.5 (20.2, 26.8)	196	32.1 (26.9, 37.3)
Adjusted prevalence difference (95% CI; q-value)[‡]	Referent		4.5 (1.6, 7.4; 0.01)		5.0 (1.4, 8.7; 0.02)		14.3 (8.7, 20.0; < 0.001)	
Participate as a combatant (someone who is fighting)								
Not likely	3812	92.1 (91, 93.1)	1736	85.6 (83.5, 87.6)	911	72.9 (69.4, 76.3)	328	62.7 (57.4, 68.0)
Somewhat likely	242	4.9 (4.1, 5.8)	254	9.9 (8.2, 11.6)	252	17.3 (14.4, 20.1)	147	21.0 (16.8, 25.3)
Very or extremely likely	60	1.3 (0.9, 1.8)	62	3.1 (2.0, 4.1)	97	8.1 (5.8, 10.3)	87	13.7 (9.9, 17.4)
Adjusted prevalence difference (95% CI; q-value)[‡]	Referent		1.0 (-0.1, 2.1; 0.12)		6.3 (4.1, 8.5; < 0.001)		11.8 (8.1, 15.5; < 0.001)	
Kill a combatant from the opposing side								
Not likely	3875	93.8 (92.9, 94.8)	1805	89.1 (87.4, 90.9)	981	77.8 (74.5, 81.1)	359	66.3 (61.2, 71.4)
Somewhat likely	171	3.2 (2.5, 3.8)	192	6.8 (5.5, 8.2)	196	12.9 (10.5, 15.4)	115	16.5 (12.8, 20.2)
Very or extremely likely	62	1.2 (0.8, 1.6)	58	2.5 (1.6, 3.5)	80	7.4 (5.0, 9.7)	85	13.7 (9.9, 17.4)
Adjusted prevalence difference (95% CI; q-value)[‡]	Referent		1.0 (0.0, 2.1; 0.08)		5.9 (3.7, 8.1; < 0.001)		12.2 (8.5, 16.0; < 0.001)	
Kill a non-combatant from the opposing side								
Not likely	4064	97.0 (96.3, 97.8)	2011	95.8 (94.4, 97.1)	1161	86.7 (83.8, 89.7)	479	82.0 (77.5, 86.4)
Somewhat likely	30	0.7 (0.4, 1.1)	25	1.9 (0.8, 2.9)	71	8.8 (6.2, 11.3)	48	7.7 (4.6, 10.8)
Very or extremely likely	14	0.4 (0.1, 0.7)	15	0.7 (0.3, 1.1)	23	2.2 (1.1, 3.3)	33	6.6 (3.8, 9.5)
Adjusted prevalence difference (95% CI; q-value)[‡]	Referent		0.2 (-0.4, 0.8; 0.6)		1.6 (0.4, 2.8; 0.02)		6.2 (3.3, 9.1; < 0.001)	

Details of categorization for beliefs about violence to effect social change are in the Additional Methods Text section of this supplement (p 13).

* Adjusted prevalence differences are for the strongly or very strongly agree comparison.

† Adjusted prevalence differences are for support of the insurgency (rather than the government or neither side).

‡ Adjusted prevalence differences are for the very or extremely likely option.

Adjusted models include age, race and ethnicity, gender, education, income, Census division, and rurality. Q-values represent the probability that the given difference would be a false discovery; they represent the expected proportion of “false positives” that would be seen among the collection of all differences whose q-values were at or below the given q-value.

Refusals were not included in the table but were included in calculating weighted percentages.

Table S9. Association between approval of extreme right-wing organizations and social movements and views on civil war in the United States

Query and Response	Approval of Organizations and Movements							
	Non-Approval		Weak Approval		Moderate Approval		Strong Approval	
	Unweighted n	Weighted % (95% CI)	Unweighted n	Weighted % (95% CI)	Unweighted n	Weighted % (95% CI)	Unweighted n	Weighted % (95% CI)
How much do you agree or disagree with each of the following statements?†								
In the next few years, there will be civil war in the United States.								
Do not agree	1765	71.3 (69.0, 73.6)	241	50.5 (44.3, 56.6)	49	30.6 (20.3, 40.9)	11	22.3 (6.0, 38.6)
Somewhat agree	612	25.1 (23.0, 27.3)	165	36.6 (30.6, 42.6)	55	45.9 (34.2, 57.6)	9	21.9 (6.7, 37.1)
Strongly or very strongly agree	61	2.9 (2.0, 3.8)	41	11.8 (7.5, 16.2)	22	22.7 (12.0, 33.4)	24	55.7 (37.0, 74.5)
Adjusted prevalence difference (95% CI; q-value)*	Referent		8.0 (3.6, 12.5; 0.003)		18.6 (8.7, 28.5; 0.002)		50.6 (32.6, 68.5; < 0.001)	
The United States needs a civil war to set things right.								
Do not agree	2358	95.2 (93.9, 96.4)	358	79.0 (74.2, 83.9)	68	43.8 (32.4, 55.3)	12	24.9 (7.9, 41.9)
Somewhat agree	68	3.5 (2.4, 4.5)	65	13.6 (9.6, 17.6)	38	32.5 (21.2, 43.8)	9	24.3 (8.2, 40.4)
Strongly or very strongly agree	17	0.8 (0.3, 1.4)	24	6.0 (3.1, 8.9)	20	22.9 (12.5, 33.4)	23	50.8 (31.9, 69.7)
Adjusted prevalence difference (95% CI; q-value)*	Referent		4.3 (1.5, 7.2; 0.01)		20.9 (10.9, 30.9; < 0.001)		47.6 (29.4, 65.8; < 0.001)	
Suppose a civil war occurred and took the form of a RIGHT-wing anti-government insurgency. Which side would you most likely support?								
Neither side	873	38.6 (36.2, 41.0)	243	60.4 (54.6, 66.2)	63	61.4 (50.7, 72.2)	15	40.4 (21.8, 58.9)
The government	1496	57.8 (55.3, 60.3)	136	26.4 (21.3, 31.5)	24	16.5 (8.6, 24.4)	14	35.7 (16.6, 54.7)
The insurgency	58	2.5 (1.7, 3.3)	68	12.3 (8.5, 16.0)	39	21.3 (13.0, 29.6)	13	19.8 (7.0, 32.6)
Adjusted prevalence difference (95% CI; q-value)†	Referent		10.6 (6.7, 14.6; < 0.001)		19.6 (11.3, 27.9; < 0.001)		20.2 (7.0, 33.5; 0.01)	
Suppose a civil war occurred and took the form of a LEFT-wing anti-government insurgency. Which side would you most likely support?								
Neither side	1121	49.3 (46.8, 51.8)	241	59.9 (54.0, 65.8)	67	65.6 (55.5, 75.8)	18	41.8 (23.3, 60.4)
The government	1106	40.2 (37.9, 42.6)	181	32.8 (27.4, 38.3)	53	30.9 (21.1, 40.8)	16	33.6 (15.6, 51.6)
The insurgency	198	9.3 (7.8, 10.8)	25	6.3 (3.1, 9.5)	6	2.7 (0.2, 5.2)	9	22.2 (6.5, 38.0)
Adjusted prevalence difference (95% CI; q-value)†	Referent		-1.3 (-4.7, 2.2; 0.58)		-7.3 (-11.3, -3.4; 0.002)		9.3 (-5.7, 24.4; 0.35)	
If a civil war did occur, how likely would you be to do each of the following?								
Leave the United States								
Not likely	1377	53.4 (50.9, 55.8)	334	67.4 (61.5, 73.4)	86	58.3 (46.5, 70.2)	23	44.6 (26.0, 63.2)
Somewhat likely	718	29.4 (27.2, 31.6)	77	18.7 (13.9, 23.6)	24	25.5 (14.2, 36.8)	6	12.7 (2.1, 23.3)
Very or extremely likely	334	16.1 (14.2, 18.0)	37	12.5 (7.9, 17.1)	17	15.6 (7.3, 23.8)	15	42.6 (23.6, 61.7)
Adjusted prevalence difference (95% CI; q-value)‡	Referent		-1.5 (-6.4, 3.4; 0.65)		-0.7 (-9.3, 7.9; 0.89)		22.2 (4.4, 39.9; 0.04)	
"Sit it out": stay in the United States, without participating in the conflict								
Not likely	293	13.1 (11.3, 14.8)	99	21.0 (15.9, 26.0)	32	27.8 (16.7, 38.9)	13	24.0 (8.9, 39.2)
Somewhat likely	886	34.6 (32.3, 36.9)	154	29.6 (24.4, 34.8)	45	37.6 (26.0, 49.2)	13	28.0 (10.1, 45.8)
Very or extremely likely	1246	51.1 (48.6, 53.5)	194	48.0 (41.9, 54.2)	50	34.0 (23.5, 44.4)	18	48.0 (29.2, 66.8)
Adjusted prevalence difference (95% CI; q-value)‡	Referent		-4.6 (-11.3, 2.2; 0.33)		-17.5 (-28.1, -7.0; 0.01)		-4.1 (-22.6, 14.5; 0.74)	
Participate in a non-combat role (examples: providing food and supplies, working as a mechanic or medic)								
Not likely	921	41.0 (38.5, 43.4)	126	30.8 (24.9, 36.7)	39	37.8 (26.1, 49.5)	11	27.9 (11.1, 44.7)
Somewhat likely	987	37.3 (34.9, 39.6)	185	37.5 (31.6, 43.3)	50	36.1 (24.7, 47.5)	10	21.1 (5.7, 36.5)
Very or extremely likely	515	20.3 (18.4, 22.3)	133	30.0 (24.4, 35.6)	36	24.3 (15.1, 33.5)	23	51.0 (32.2, 69.9)
Adjusted prevalence difference (95% CI; q-value)‡	Referent		8.3 (2.5, 14.2; 0.02)		3.3 (-6.0, 12.7; 0.58)		29.2 (10.8, 47.7; 0.01)	
Participate as a combatant (someone who is fighting)								
Not likely	2205	90.2 (88.6, 91.7)	338	75.5 (70.3, 80.6)	77	67.4 (57.1, 77.6)	15	44.4 (25.5, 63.3)
Somewhat likely	177	6.9 (5.6, 8.1)	71	14.9 (10.9, 19.0)	26	14.4 (7.7, 21.1)	11	16.1 (3.8, 28.5)
Very or extremely likely	45	1.8 (1.1, 2.5)	36	7.8 (4.4, 11.1)	22	16.6 (8.2, 25.1)	17	39.0 (20.6, 57.4)
Adjusted prevalence difference (95% CI; q-value)‡	Referent		5.4 (1.9, 8.9; 0.01)		12.6 (4.8, 20.3; 0.01)		34.2 (18.0, 50.4; < 0.001)	
Kill a combatant from the opposing side								
Not likely	2253	92.3 (91.0, 93.7)	353	79.7 (75.0, 84.4)	86	71.8 (62.0, 81.6)	20	50.2 (31.4, 69.1)
Somewhat likely	125	4.0 (3.2, 4.9)	63	12.2 (8.6, 15.8)	23	15.7 (7.8, 23.6)	7	12.7 (1.3, 24.0)
Very or extremely likely	45	2.2 (1.3, 3.1)	29	6.5 (3.4, 9.6)	17	11.2 (4.7, 17.7)	17	37.1 (18.8, 55.4)
Adjusted prevalence difference (95% CI; q-value)‡	Referent		4.1 (0.8, 7.5; 0.04)		7.3 (0.6, 14.0; 0.08)		32.4 (15.7, 49.1; 0.001)	
Kill a non-combatant from the opposing side								
Not likely	2382	96.3 (95.2, 97.4)	421	91.5 (87.8, 95.2)	104	79.1 (69.7, 88.4)	25	57.2 (38.4, 75.9)
Somewhat likely	29	1.7 (0.9, 2.5)	16	3.8 (1.4, 6.2)	12	13.7 (5.2, 22.3)	10	25.5 (7.5, 43.6)
Very or extremely likely	13	0.7 (0.2, 1.2)	8	2.6 (0.3, 5.0)	9	5.7 (1.5, 9.9)	9	17.3 (5.1, 29.5)
Adjusted prevalence difference (95% CI; q-value)‡	Referent		1.7 (-0.7, 4.0; 0.3)		4.1 (-0.5, 8.7; 0.18)		15.4 (3.8, 27.0; 0.03)	

Organizations were the Proud Boys, Oath Keepers, and Three Percenters; social movements were the QAnon, white supremacy, Christian nationalist, militia, and boogaloo movements.

Details of categorization for approval of organizations and movements are in the Additional Methods Text section of this supplement (p 13).

* Adjusted prevalence differences are for the strongly or very strongly agree comparison.

† Adjusted prevalence differences are for support of the insurgency (rather than the government or neither side).

‡ Adjusted prevalence differences are for the very or extremely likely option.

Adjusted models include age, race and ethnicity, gender, education, income, Census division, and rurality. Q-values represent the probability that the given difference would be a false discovery; they represent the expected proportion of “false positives” that would be seen among the collection of all differences whose q-values were at or below the given q-value.

Refusals were not included in the table but were included in calculating weighted percentages.

Table S10. Association between firearm ownership status and views on civil war in the United States

Query and Response	Firearm Ownership Status					
	Nonowner without Firearms at Home		Nonowner with Firearms at Home		Owner	
	Unweighted n	Weighted % (95% CI)	Unweighted n	Weighted % (95% CI)	Unweighted n	Weighted % (95% CI)
How much do you agree or disagree with each of the following statements?[†]						
In the next few years, there will be civil war in the United States.						
Do not agree	2441	63.1 (61.1, 65.1)	324	55.8 (50.7, 60.9)	2488	61.9 (59.8, 63.9)
Somewhat agree	1016	27.7 (25.9, 29.6)	190	35.9 (31.0, 40.8)	1122	30.3 (28.4, 32.3)
Strongly or very strongly agree	187	6.6 (5.5, 7.7)	23	6.0 (3.0, 9.0)	217	6.8 (5.7, 7.9)
Adjusted prevalence difference (95% CI; q-value)*	Referent		-0.2 (-3.4, 3.1; 0.93)		1.7 (0, 3.3; 0.16)	
The United States needs a civil war to set things right.						
Do not agree	3309	85.7 (84.1, 87.2)	483	86.5 (82.9, 90.1)	3287	83.5 (81.9, 85.0)
Somewhat agree	250	8.4 (7.2, 9.6)	46	8.9 (6.0, 11.8)	409	11.1 (9.8, 12.3)
Strongly or very strongly agree	90	3.5 (2.7, 4.3)	8	1.9 (0.2, 3.6)	133	4.6 (3.6, 5.6)
Adjusted prevalence difference (95% CI; q-value)*	Referent		-0.8 (-2.7, 1.2; 0.57)		2.2 (0.8, 3.6; 0.01)	
Suppose a civil war occurred and took the form of a RIGHT-wing anti-government insurgency. Which side would you most likely support?						
Neither side	1795	54.7 (52.7, 56.7)	295	56.7 (51.7, 61.6)	1772	50.3 (48.3, 52.4)
The government	1621	37.1 (35.2, 39.0)	206	33.5 (28.8, 38.2)	1513	33.0 (31.2, 34.9)
The insurgency	202	5.3 (4.4, 6.2)	34	7.1 (4.3, 9.9)	516	15.0 (13.4, 16.5)
Adjusted prevalence difference (95% CI; q-value)[†]	Referent		1.7 (-1.5, 4.8; 0.5)		8.9 (6.9, 10.8; < 0.001)	
Suppose a civil war occurred and took the form of a LEFT-wing anti-government insurgency. Which side would you most likely support?						
Neither side	2018	60.0 (58, 61.9)	302	57.0 (52.1, 62.0)	1827	53.0 (50.9, 55.0)
The government	1352	30.1 (28.4, 31.9)	201	32.6 (28.1, 37.2)	1762	38.9 (37.0, 40.8)
The insurgency	241	6.7 (5.7, 7.7)	32	7.9 (4.7, 11.1)	209	6.3 (5.3, 7.3)
Adjusted prevalence difference (95% CI; q-value)[†]	Referent		1.0 (-2.3, 4.3; 0.67)		0.6 (-0.9, 2.1; 0.57)	
If a civil war did occur, how likely would you be to do each of the following?						
Leave the United States						
Not likely	2214	56.0 (54.0, 58.1)	352	60.1 (55.1, 65.2)	2838	71.8 (70.0, 73.7)
Somewhat likely	964	26.9 (25.1, 28.8)	144	27.7 (23.1, 32.4)	698	18.9 (17.3, 20.5)
Very or extremely likely	461	14.3 (12.9, 15.8)	46	11.2 (7.7, 14.7)	297	8.5 (7.3, 9.6)
Adjusted prevalence difference (95% CI; q-value)[‡]	Referent		-3.2 (-7.1, 0.6; 0.28)		-3.5 (-5.4, -1.5; 0.01)	
"Sit it out": stay in the United States, without participating in the conflict						
Not likely	516	15.6 (14.1, 17.1)	55	9.9 (6.9, 12.9)	599	16.4 (14.7, 18.0)
Somewhat likely	1283	33.7 (31.8, 35.6)	196	35.9 (31.1, 40.7)	1456	36.6 (34.7, 38.5)
Very or extremely likely	1836	48.1 (46.0, 50.1)	292	53.3 (48.3, 58.3)	1761	45.9 (43.8, 47.9)
Adjusted prevalence difference (95% CI; q-value)[‡]	Referent		4.0 (-1.6, 9.6; 0.35)		-3.9 (-7.0, -0.7; 0.08)	
Participate in a non-combat role (examples: providing food and supplies, working as a mechanic or medic)						
Not likely	1416	41.0 (39.0, 43.0)	178	35.1 (30.2, 40.0)	1262	33.8 (31.9, 35.8)
Somewhat likely	1416	35.1 (33.2, 37.0)	245	42.6 (37.7, 47.6)	1642	41.2 (39.2, 43.2)
Very or extremely likely	789	20.8 (19.2, 22.4)	117	21.0 (16.9, 25.1)	908	23.6 (21.9, 25.3)
Adjusted prevalence difference (95% CI; q-value)[‡]	Referent		0.3 (-4.4, 4.9; 0.93)		3.1 (0.5, 5.7; 0.1)	
Participate as a combatant (someone who is fighting)						
Not likely	3289	86.8 (85.4, 88.3)	508	91.2 (88.1, 94.3)	2986	76.1 (74.3, 77.9)
Somewhat likely	252	7.4 (6.3, 8.6)	27	5.1 (2.8, 7.3)	613	16.0 (14.6, 17.5)
Very or extremely likely	82	2.8 (2.0, 3.5)	6	2.5 (0.3, 4.7)	215	6.7 (5.5, 7.8)
Adjusted prevalence difference (95% CI; q-value)[‡]	Referent		1.2 (-1.0, 3.5; 0.49)		4.5 (3.0, 6.0; < 0.001)	
Kill a combatant from the opposing side						
Not likely	3380	89.9 (88.6, 91.2)	506	91.7 (88.7, 94.7)	3134	80.0 (78.4, 81.7)
Somewhat likely	174	4.8 (3.9, 5.7)	22	4.1 (2.1, 6.1)	474	12.5 (11.2, 13.8)
Very or extremely likely	73	2.4 (1.7, 3.1)	10	2.9 (0.8, 5.1)	198	6.1 (5.0, 7.2)
Adjusted prevalence difference (95% CI; q-value)[‡]	Referent		1.9 (-0.4, 4.2; 0.3)		4.1 (2.6, 5.6; < 0.001)	
Kill a non-combatant from the opposing side						
Not likely	3520	93.1 (91.9, 94.2)	522	94.0 (91.2, 96.7)	3662	93.7 (92.6, 94.8)
Somewhat likely	62	2.6 (1.8, 3.4)	15	4.2 (1.7, 6.6)	99	3.2 (2.3, 4.0)
Very or extremely likely	35	1.2 (0.7, 1.7)	3	0.7 (0.0, 1.7)	47	1.4 (0.9, 1.9)
Adjusted prevalence difference (95% CI; q-value)[‡]	Referent		-0.1 (-1.2, 1.0; 0.92)		0.7 (0.0, 1.4; 0.2)	

Details of categorization for firearm ownership status are in the Additional Methods Text section of this supplement (p 13).

* Adjusted prevalence differences are for the strongly or very strongly agree comparison.

† Adjusted prevalence differences are for support of the insurgency (rather than the government or neither side).

‡ Adjusted prevalence differences are for the very or extremely likely option.

Adjusted models include age, race and ethnicity, gender, education, income, Census division, and rurality. Q-values represent the probability that the given difference would be a false discovery; they represent the expected proportion of "false positives" that would be seen among the collection of all differences whose q-values were at or below the given q-value.

Refusals were not included in the table but were included in calculating weighted percentages.

Table S11. Association between types(s) of firearm owned and views on civil war in the United States

Query and Response	Type(s) of Firearm Owned							
	Handgun Only		Other		Other Rifle		Assault-Type Rifle	
	Unweighted n	Weighted % (95% CI)	Unweighted n	Weighted % (95% CI)	Unweighted n	Weighted % (95% CI)	Unweighted n	Weighted % (95% CI)
How much do you agree or disagree with each of the following statements?[†]								
In the next few years, there will be civil war in the United States.								
Do not agree	569	60.0 (56.0, 64.1)	325	65.5 (60.3, 70.7)	1126	63.7 (60.5, 66.8)	419	58.4 (53.6, 63.1)
Somewhat agree	271	32.1 (28.2, 36.0)	131	24.9 (20.3, 29.4)	486	31.2 (28.1, 34.3)	210	31.0 (26.5, 35.5)
Strongly or very strongly agree	56	7.2 (5.0, 9.5)	31	9.0 (5.6, 12.5)	68	4.1 (3.0, 5.2)	56	10.0 (7.0, 13.1)
Adjusted prevalence difference (95% CI; q-value)*	Referent		2.9 (-1.3, 7.2; 0.53)		-2.1 (-4.9, 0.7; 0.53)		4.0 (0, 8.1; 0.43)	
The United States needs a civil war to set things right.								
Do not agree	778	84.2 (81.1, 87.2)	431	87.5 (84.0, 91.0)	1477	86.7 (84.6, 88.7)	537	76.4 (72.3, 80.5)
Somewhat agree	93	10.4 (8.1, 12.7)	41	8.2 (5.4, 10.9)	164	10.0 (8.2, 11.9)	100	14.5 (11.2, 17.9)
Strongly or very strongly agree	26	4.8 (2.6, 7.0)	16	4.1 (1.7, 6.4)	41	2.7 (1.8, 3.6)	45	7.9 (5.2, 10.5)
Adjusted prevalence difference (95% CI; q-value)*	Referent		-0.6 (-4.0, 2.8; 0.86)		-1.9 (-4.5, 0.8; 0.53)		3.1 (-0.8, 7.1; 0.53)	
Suppose a civil war occurred and took the form of a RIGHT-wing anti-government insurgency. Which side would you most likely support?								
Neither side	432	52.7 (48.6, 56.8)	217	50.5 (45.1, 56.0)	754	48.2 (45.1, 51.4)	324	49.5 (44.8, 54.2)
The government	357	32.7 (29.0, 36.3)	211	36.8 (31.8, 41.8)	708	36.4 (33.4, 39.3)	214	26.4 (22.6, 30.1)
The insurgency	102	13.1 (10.1, 16.0)	51	10.9 (7.5, 14.2)	211	14.1 (11.7, 16.4)	141	22.5 (18.2, 26.8)
Adjusted prevalence difference (95% CI; q-value)[†]	Referent		-3.6 (-8.4, 1.2; 0.53)		-1.3 (-5.5, 3.0; 0.83)		5.4 (0.1, 10.8; 0.43)	
Suppose a civil war occurred and took the form of a LEFT-wing anti-government insurgency. Which side would you most likely support?								
Neither side	440	54.2 (50.1, 58.2)	225	53.6 (48.2, 59.0)	786	51.4 (48.3, 54.6)	329	52.8 (48.1, 57.4)
The government	392	36.6 (32.9, 40.4)	232	39.8 (34.6, 44.9)	804	41.5 (38.4, 44.6)	305	38.4 (33.9, 42.8)
The insurgency	60	7.5 (5.2, 9.7)	24	5.3 (2.8, 7.9)	80	5.7 (4.1, 7.2)	42	6.5 (4.2, 8.7)
Adjusted prevalence difference (95% CI; q-value)[†]	Referent		-2.6 (-6.2, 1.0; 0.53)		-1.8 (-5.1, 1.4; 0.62)		-2.7 (-6.4, 1.0; 0.53)	
If a civil war did occur, how likely would you be to do each of the following?								
Leave the United States								
Not likely	624	66.2 (62.1, 70.2)	332	64.5 (59.2, 69.9)	1293	75.3 (72.6, 78.0)	524	75.2 (71.3, 79.1)
Somewhat likely	188	23.1 (19.3, 26.8)	113	25.8 (20.7, 30.8)	271	15.7 (13.5, 17.8)	114	17.2 (13.8, 20.6)
Very or extremely likely	87	10.3 (7.8, 12.7)	41	9.1 (6.0, 12.2)	118	8.1 (6.3, 9.9)	47	7.4 (5.0, 9.8)
Adjusted prevalence difference (95% CI; q-value)[‡]	Referent		0.5 (-3.5, 4.5; 0.88)		0.9 (-2.3, 4.1; 0.83)		-2.0 (-5.7, 1.7; 0.63)	
"Sit it out": stay in the United States, without participating in the conflict								
Not likely	122	14.5 (11.6, 17.5)	68	13.0 (9.5, 16.6)	252	15.2 (12.7, 17.7)	145	22.8 (18.5, 27.2)
Somewhat likely	331	36.6 (32.7, 40.6)	175	36.0 (30.7, 41.3)	635	36.1 (33.2, 39.0)	276	38.4 (33.9, 42.9)
Very or extremely likely	441	47.8 (43.7, 51.9)	241	50.1 (44.6, 55.5)	790	47.5 (44.3, 50.7)	261	38.1 (33.6, 42.6)
Adjusted prevalence difference (95% CI; q-value)[‡]	Referent		3.1 (-3.8, 10.1; 0.73)		1.5 (-3.9, 6.9; 0.84)		-8.4 (-15.0, -1.9; 0.17)	
Participate in a non-combat role (examples: providing food and supplies, working as a mechanic or medic)								
Not likely	303	33.1 (29.3, 36.9)	170	37.2 (31.7, 42.7)	549	33.3 (30.3, 36.3)	214	33.8 (29.1, 38.5)
Somewhat likely	365	39.5 (35.5, 43.5)	195	37.7 (32.5, 43.0)	732	42.6 (39.4, 45.7)	309	43.4 (38.8, 48.0)
Very or extremely likely	224	26.1 (22.4, 29.8)	117	23.7 (19.2, 28.2)	394	22.7 (20.2, 25.3)	161	22.5 (18.8, 26.2)
Adjusted prevalence difference (95% CI; q-value)[‡]	Referent		0.2 (-5.5, 5.8; 0.96)		-0.4 (-4.9, 4.1; 0.89)		0.9 (-4.5, 6.2; 0.86)	
Participate as a combatant (someone who is fighting)								
Not likely	760	82.9 (79.7, 86.1)	393	78.5 (74.0, 82.9)	1335	78.0 (75.4, 80.6)	438	63.2 (58.7, 67.7)
Somewhat likely	97	11.2 (8.6, 13.8)	65	14.2 (10.5, 17.9)	269	15.5 (13.4, 17.6)	168	23.9 (20.0, 27.8)
Very or extremely likely	35	4.7 (2.7, 6.6)	24	6.1 (3.4, 8.9)	74	5.2 (3.6, 6.9)	76	12.4 (9.3, 15.6)
Adjusted prevalence difference (95% CI; q-value)[‡]	Referent		1.7 (-1.7, 5.0; 0.7)		1.9 (-0.7, 4.6; 0.53)		7.4 (3.6, 11.2; 0.01)	
Kill a combatant from the opposing side								
Not likely	789	85.9 (82.9, 89.0)	414	83.6 (79.7, 87.6)	1399	82.1 (79.7, 84.5)	467	66.1 (61.7, 70.5)
Somewhat likely	69	7.8 (5.6, 9.9)	51	11.6 (8.1, 15.1)	198	11.5 (9.6, 13.3)	147	22.4 (18.5, 26.2)
Very or extremely likely	32	4.9 (2.7, 7.1)	18	3.8 (1.9, 5.8)	75	4.8 (3.4, 6.2)	67	10.9 (7.9, 13.9)
Adjusted prevalence difference (95% CI; q-value)[‡]	Referent		-1.6 (-4.4, 1.2; 0.62)		0.5 (-2.0, 2.9; 0.86)		5.3 (1.5, 9.1; 0.14)	
Kill a non-combatant from the opposing side								
Not likely	868	95.5 (93.8, 97.2)	462	93.6 (90.6, 96.6)	1632	95.3 (93.7, 96.9)	625	90.0 (87.3, 92.8)
Somewhat likely	15	1.7 (0.7, 2.8)	16	3.3 (1.5, 5.1)	31	2.4 (1.2, 3.5)	33	5.5 (3.4, 7.6)
Very or extremely likely	9	1.3 (0.3, 2.2)	4	0.8 (0.0, 1.7)	11	0.7 (0.1, 1.3)	22	3.5 (1.8, 5.2)
Adjusted prevalence difference (95% CI; q-value)[‡]	Referent		0.1 (-1.2, 1.4; 0.89)		0.4 (-0.7, 1.5; 0.8)		2.7 (0.7, 4.6; 0.14)	

Details of categorization for firearm owners by type(s) of firearms owned are in the Additional Methods Text section of this supplement (p 13).

* Adjusted prevalence differences are for the strongly or very strongly agree comparison.

† Adjusted prevalence differences are for support of the insurgency (rather than the government or neither side).

‡ Adjusted prevalence differences are for the very or extremely likely option.

Adjusted models include age, race and ethnicity, gender, education, income, Census division, and rurality. Q-values represent the probability that the given difference would be a false discovery; they represent the expected proportion of "false positives" that would be seen among the collection of all differences whose q-values were at or below the given q-value.

Refusals were not included in the table but were included in calculating weighted percentages.

Table S12. Association between recency of firearm purchase and views on civil war in the United States

Query and Response	Recency of Firearm Purchase			
	Purchases Only 2019 or Earlier		Purchases 2020 or Later	
	Unweighted n	Weighted % (95% CI)	Unweighted n	Weighted % (95% CI)
How much do you agree or disagree with each of the following statements?†				
In the next few years, there will be civil war in the United States.				
Do not agree	1819	64.1 (61.7, 66.6)	636	56.7 (52.9, 60.5)
Somewhat agree	762	29.2 (26.9, 31.5)	345	33.0 (29.5, 36.6)
Strongly or very strongly agree	129	5.6 (4.4, 6.7)	86	9.7 (7.3, 12.1)
Adjusted prevalence difference (95% CI; q-value)*	Referent		3.8 (1.3, 6.3; 0.02)	
The United States needs a civil war to set things right.				
Do not agree	2390	85.5 (83.6, 87.3)	859	79.5 (76.6, 82.5)
Somewhat agree	256	10.2 (8.7, 11.8)	144	12.9 (10.6, 15.2)
Strongly or very strongly agree	68	3.6 (2.5, 4.7)	62	6.8 (4.8, 8.9)
Adjusted prevalence difference (95% CI; q-value)*	Referent		2.5 (0.1, 4.8; 0.07)	
Suppose a civil war occurred and took the form of a RIGHT-wing anti-government insurgency. Which side would you most likely support?				
Neither side	1216	49.7 (47.2, 52.1)	530	51.6 (47.8, 55.4)
The government	1163	36.1 (33.8, 38.3)	338	27.4 (24.1, 30.7)
The insurgency	314	12.6 (10.9, 14.4)	192	19.6 (16.5, 22.8)
Adjusted prevalence difference (95% CI; q-value)†	Referent		6.6 (2.9, 10.2; 0.01)	
Suppose a civil war occurred and took the form of a LEFT-wing anti-government insurgency. Which side would you most likely support?				
Neither side	1270	52.7 (50.2, 55.1)	530	53.6 (49.8, 57.3)
The government	1283	40.4 (38.0, 42.7)	460	36.4 (32.8, 40.0)
The insurgency	138	5.3 (4.3, 6.3)	69	8.3 (5.9, 10.7)
Adjusted prevalence difference (95% CI; q-value)†	Referent		1.9 (-0.6, 4.4; 0.21)	
If a civil war did occur, how likely would you be to do each of the following?				
Leave the United States				
Not likely	2003	72.0 (69.8, 74.2)	794	71.2 (67.7, 74.7)
Somewhat likely	509	19.4 (17.5, 21.3)	183	18.3 (15.3, 21.3)
Very or extremely likely	202	7.7 (6.4, 8.9)	91	10.1 (7.7, 12.5)
Adjusted prevalence difference (95% CI; q-value)‡	Referent		0.8 (-1.8, 3.4; 0.6)	
"Sit it out": stay in the United States, without participating in the conflict				
Not likely	371	14.1 (12.2, 15.9)	217	21.0 (17.7, 24.3)
Somewhat likely	1037	37.0 (34.7, 39.3)	399	35.6 (32.1, 39.2)
Very or extremely likely	1294	47.7 (45.3, 50.2)	448	42.4 (38.7, 46.2)
Adjusted prevalence difference (95% CI; q-value)‡	Referent		-5.8 (-10.4, -1.3; 0.04)	
Participate in a non-combat role (examples: providing food and supplies, working as a mechanic or medic)				
Not likely	908	35.0 (32.7, 37.4)	339	31.6 (28.1, 35.2)
Somewhat likely	1168	41.2 (38.8, 43.6)	450	41.0 (37.3, 44.8)
Very or extremely likely	626	22.4 (20.5, 24.4)	272	26.3 (22.9, 29.6)
Adjusted prevalence difference (95% CI; q-value)‡	Referent		4.3 (0.4, 8.2; 0.07)	
Participate as a combatant (someone who is fighting)				
Not likely	2221	80.3 (78.4, 82.3)	729	67.3 (63.8, 70.9)
Somewhat likely	374	13.6 (11.9, 15.2)	230	21.1 (18.0, 24.1)
Very or extremely likely	105	4.8 (3.7, 5.9)	105	10.7 (8.2, 13.2)
Adjusted prevalence difference (95% CI; q-value)‡	Referent		4.4 (1.7, 7.1; 0.01)	
Kill a combatant from the opposing side				
Not likely	2307	83.4 (81.5, 85.3)	785	72.4 (69.0, 75.7)
Somewhat likely	287	10.5 (9.1, 11.9)	184	17.2 (14.4, 20.0)
Very or extremely likely	100	4.6 (3.3, 5.9)	93	9.4 (7.2, 11.6)
Adjusted prevalence difference (95% CI; q-value)‡	Referent		3.5 (0.9, 6.2; 0.04)	
Kill a non-combatant from the opposing side				
Not likely	2620	94.7 (93.4, 96.1)	993	91.5 (89.4, 93.6)
Somewhat likely	56	2.8 (1.7, 3.8)	42	4.2 (2.7, 5.6)
Very or extremely likely	22	0.8 (0.4, 1.1)	25	2.9 (1.6, 4.3)
Adjusted prevalence difference (95% CI; q-value)‡	Referent		1.6 (0.3, 2.9; 0.04)	

Details of categorization for firearm owners by recency of purchase are in the Additional Methods Text section of this supplement (p 13).

* Adjusted prevalence differences are for the strongly or very strongly agree comparison.

† Adjusted prevalence differences are for support of the insurgency (rather than the government or neither side).

‡ Adjusted prevalence differences are for the very or extremely likely option.

Adjusted models include age, race and ethnicity, gender, education, income, Census division, and rurality. Q-values represent the probability that the given difference would be a false discovery; they represent the expected proportion of “false positives” that would be seen among the collection of all differences whose q-values were at or below the given q-value.

Refusals were not included in the table but were included in calculating weighted percentages.

Table S13. Association between frequency of firearm carrying and views on civil war in the United States

Query and Response	Carrying Loaded Firearm When Out in Public in the Past Year					
	Never or Not Often at All		Less Than Half, About Half, or More Than Half the Time		All or Nearly All the Time	
	Unweighted n	Weighted % (95% CI)	Unweighted n	Weighted % (95% CI)	Unweighted n	Weighted % (95% CI)
How much do you agree or disagree with each of the following statements?†						
In the next few years, there will be civil war in the United States.						
Do not agree	2020	64.3 (62.1, 66.5)	313	58.4 (53.1, 63.8)	146	46.9 (39.4, 54.4)
Somewhat agree	843	29.1 (27.0, 31.3)	163	31.3 (26.3, 36.2)	112	38.6 (30.7, 46.5)
Strongly or very strongly agree	139	5.7 (4.5, 6.8)	40	8.9 (5.8, 12.1)	37	13.0 (8.1, 17.9)
Adjusted prevalence difference (95% CI; q-value)*	Referent		3.5 (0.2, 6.9; 0.1)		6.7 (1.6, 11.8; 0.03)	
The United States needs a civil war to set things right.						
Do not agree	2661	86.0 (84.3, 87.7)	407	78.6 (74.2, 83.0)	207	69.5 (62.6, 76.4)
Somewhat agree	268	9.6 (8.3, 11.0)	81	13.8 (10.4, 17.3)	59	19.3 (13.3, 25.3)
Strongly or very strongly agree	75	3.6 (2.5, 4.6)	29	7.1 (4.0, 10.1)	28	9.5 (5.5, 13.6)
Adjusted prevalence difference (95% CI; q-value)*	Referent		3.3 (0.2, 6.5; 0.1)		5.2 (0.8, 9.6; 0.06)	
Suppose a civil war occurred and took the form of a RIGHT-wing anti-government insurgency. Which side would you most likely support?						
Neither side	1376	50.1 (47.8, 52.4)	250	50.5 (45.1, 55.8)	138	51.4 (43.8, 59.0)
The government	1280	36.3 (34.2, 38.5)	152	24.9 (20.7, 29.2)	77	19.5 (14.6, 24.4)
The insurgency	324	11.9 (10.3, 13.5)	109	22.8 (17.9, 27.7)	82	29.0 (22.1, 35.8)
Adjusted prevalence difference (95% CI; q-value)†	Referent		10.1 (5.0, 15.2; < 0.001)		17.0 (10.5, 23.5; < 0.001)	
Suppose a civil war occurred and took the form of a LEFT-wing anti-government insurgency. Which side would you most likely support?						
Neither side	1423	52.5 (50.2, 54.8)	247	53.6 (48.3, 58.9)	149	55.3 (47.8, 62.8)
The government	1407	40.1 (37.9, 42.3)	230	36.4 (31.6, 41.3)	120	34.0 (27.1, 40.8)
The insurgency	152	5.9 (4.7, 7.0)	32	7.9 (4.8, 10.9)	25	7.7 (4.2, 11.1)
Adjusted prevalence difference (95% CI; q-value)†	Referent		1.1 (-2.3, 4.5; 0.62)		1.2 (-2.6, 5.0; 0.62)	
If a civil war did occur, how likely would you be to do each of the following?						
Leave the United States						
Not likely	2182	70.6 (68.5, 72.7)	404	75.2 (70.4, 79.9)	243	78.1 (71.7, 84.4)
Somewhat likely	583	20.0 (18.1, 21.9)	77	15.9 (12.0, 19.7)	34	14.2 (8.7, 19.8)
Very or extremely likely	241	8.7 (7.4, 10.0)	34	7.4 (4.5, 10.3)	21	7.7 (3.8, 11.5)
Adjusted prevalence difference (95% CI; q-value)‡	Referent		-2.0 (-5.2, 1.2; 0.36)		-1.9 (-5.9, 2.2; 0.51)	
"Sit it out": stay in the United States, without participating in the conflict						
Not likely	415	14.8 (12.9, 16.6)	109	21.2 (16.7, 25.8)	73	21.6 (16.1, 27.0)
Somewhat likely	1142	36.5 (34.3, 38.6)	193	35.0 (30.1, 39.9)	113	39.0 (31.6, 46.4)
Very or extremely likely	1436	47.7 (45.4, 50.0)	212	41.8 (36.5, 47.1)	109	38.3 (30.6, 46.0)
Adjusted prevalence difference (95% CI; q-value)‡	Referent		-5.6 (-11.5, 0.3; 0.14)		-9.2 (-16.8, -1.7; 0.05)	
Participate in a non-combat role (examples: providing food and supplies, working as a mechanic or medic)						
Not likely	1026	35.0 (32.7, 37.2)	149	30.7 (25.6, 35.8)	83	29.2 (22.1, 36.2)
Somewhat likely	1276	40.9 (38.7, 43.2)	229	41.1 (35.9, 46.3)	129	43.1 (35.4, 50.8)
Very or extremely likely	685	22.8 (20.8, 24.7)	136	26.3 (21.6, 31.0)	86	27.7 (21.5, 33.9)
Adjusted prevalence difference (95% CI; q-value)‡	Referent		5.1 (0.0, 10.2; 0.11)		5.3 (-1.4, 11.9; 0.23)	
Participate as a combatant (someone who is fighting)						
Not likely	2478	80.6 (78.8, 82.5)	326	61.1 (55.8, 66.4)	172	62.3 (55.4, 69.2)
Somewhat likely	400	13.6 (12.0, 15.1)	133	25.6 (21.0, 30.2)	77	20.9 (15.7, 26.1)
Very or extremely likely	112	4.6 (3.5, 5.7)	53	11.1 (7.5, 14.8)	49	16.8 (11.7, 22.0)
Adjusted prevalence difference (95% CI; q-value)‡	Referent		5.7 (1.7, 9.6; 0.03)		10.6 (5.4, 15.8; < 0.001)	
Kill a combatant from the opposing side						
Not likely	2581	84.7 (83.1, 86.4)	360	66.8 (61.5, 72.0)	183	61.7 (54.6, 68.8)
Somewhat likely	300	10.0 (8.7, 11.3)	103	19.0 (15.0, 23.0)	68	22.8 (16.9, 28.8)
Very or extremely likely	100	3.9 (2.8, 4.9)	50	12.1 (8.0, 16.3)	47	15.4 (10.5, 20.3)
Adjusted prevalence difference (95% CI; q-value)‡	Referent		7.5 (3.3, 11.7; 0.003)		10.4 (5.4, 15.3; < 0.001)	
Kill a non-combatant from the opposing side						
Not likely	2908	95.5 (94.4, 96.6)	468	87.4 (83.4, 91.4)	272	89.1 (84.2, 94.0)
Somewhat likely	63	2.6 (1.7, 3.5)	25	5.7 (3.1, 8.3)	11	4.0 (1.2, 6.8)
Very or extremely likely	18	0.6 (0.3, 0.9)	17	3.7 (1.5, 5.9)	12	4.8 (1.7, 7.8)
Adjusted prevalence difference (95% CI; q-value)‡	Referent		3.0 (0.8, 5.1; 0.03)		4.0 (1.0, 7.1; 0.03)	

Details on categorization for firearm owners by frequency of carrying are in the Additional Methods Text section of this supplement (p 13).

* Adjusted prevalence differences are for the strongly or very strongly agree comparison.

† Adjusted prevalence differences are for support of the insurgency (rather than the government or neither side).

‡ Adjusted prevalence differences are for the very or extremely likely option.

Adjusted models include age, race and ethnicity, gender, education, income, Census division, and rurality. Q-values represent the probability that the given difference would be a false discovery; they represent the expected proportion of “false positives” that would be seen among the collection of all differences whose q-values were at or below the given q-value.

Refusals were not included in the table but were included in calculating weighted percentages.
